# SuperSAGE: the drought stress-responsive transcriptome of chickpea roots

**DOI:** 10.1186/1471-2164-9-553

**Published:** 2008-11-24

**Authors:** Carlos Molina, Björn Rotter, Ralf Horres, Sripada M Udupa, Bert Besser, Luis Bellarmino, Michael Baum, Hideo Matsumura, Ryohei Terauchi, Günter Kahl, Peter Winter

**Affiliations:** 1 Biocenter, Frankfurt University, Max-von-Laue-Str. 9, 60439 Frankfurt am Main, Germany; 2 GenXPro GmbH, Altenhöferallee 3, 60438 Frankfurt am Main, Germany; 3 Universidade Federal de Pernambuco, UFPE, CCB, Laboratorio de Genética, Av. Prof. Moraes Rego, s/no. 50732-970, Recife – PE, Brazil; 4 ICARDA, P.O.Box 5433, Aleppo, Syria; 5 Iwate Biotechnology Research Centre (IBRC), Kitakami, Iwate, Japan; 6 Current address : INRA, Unite Mixte de Recherche en Génétique et Ecophysiologie des Légumineuses, Domaine d'Epoisses, 21110 Bretenières, France

## Abstract

**Background:**

Drought is the major constraint to increase yield in chickpea (*Cicer arietinum*). Improving drought tolerance is therefore of outmost importance for breeding. However, the complexity of the trait allowed only marginal progress. A solution to the current stagnation is expected from innovative molecular tools such as transcriptome analyses providing insight into stress-related gene activity, which combined with molecular markers and expression (e)QTL mapping, may accelerate knowledge-based breeding. SuperSAGE, an improved version of the serial analysis of gene expression (SAGE) technique, generating genome-wide, high-quality transcription profiles from any eukaryote, has been employed in the present study. The method produces 26 bp long fragments (26 bp tags) from defined positions in cDNAs, providing sufficient sequence information to unambiguously characterize the mRNAs. Further, SuperSAGE tags may be immediately used to produce microarrays and probes for real-time-PCR, thereby overcoming the lack of genomic tools in non-model organisms.

**Results:**

We applied SuperSAGE to the analysis of gene expression in chickpea roots in response to drought. To this end, we sequenced 80,238 26 bp tags representing 17,493 unique transcripts (UniTags) from drought-stressed and non-stressed control roots. A total of 7,532 (43%) UniTags were more than 2.7-fold differentially expressed, and 880 (5.0%) were regulated more than 8-fold upon stress. Their large size enabled the unambiguous annotation of 3,858 (22%) UniTags to genes or proteins in public data bases and thus to stress-response processes. We designed a microarray carrying 3,000 of these 26 bp tags. The chip data confirmed 79% of the tag-based results, whereas RT-PCR confirmed the SuperSAGE data in all cases.

**Conclusion:**

This study represents the most comprehensive analysis of the drought-response transcriptome of chickpea available to date. It demonstrates that – inter alias – signal transduction, transcription regulation, osmolyte accumulation, and ROS scavenging undergo strong transcriptional remodelling in chickpea roots already 6 h after drought stress. Certain transcript isoforms characterizing these processes are potential targets for breeding for drought tolerance. We demonstrate that these can be easily accessed by micro-arrays and RT-PCR assays readily produced downstream of SuperSAGE. Our study proves that SuperSAGE owns potential for molecular breeding also in non-model crops.

## Background

Chickpea (*Cicer arietinum *L.) is one of the most important grain legume crops worldwide and a major source of protein for millions of families in developing countries. Despite considerable investment in breeding, average chickpea yield in major producer countries such as India stagnates at 0.6–0.7 Mt hectare^-1 ^since many years. This low yield is far below the crop's potential of 3–5 Mt hectar^-1 ^under optimal conditions. Chickpea is mostly grown in low-input, rain-fed agriculture in Mediterranean-type environments as inter-crop between cereals, and on residual moisture from monsoon rains on the Indian subcontinent. Due to insufficient, untimely and erratic rainfall in these semi-arid and arid areas, the crop often suffers from drought at the end of the cropping season. In future, global warming and soil erosion will even worsen conditions for many crops including chickpea. Thus, drought represents the major constraint to increase chickpea yield, and drought tolerance therefore is a major aim of chickpea breeding. However, drought tolerance is a complex trait and hard to achieve by conventional breeding methods. Understanding of the molecular mechanisms underlying drought tolerance is therefore needed for successful, knowledge-based crop improvement [1].

Molecular genetics and genomics of stress-responses in model plants such as *Arabidopsis *revealed that abiotic stresses such as drought, salinity and cold stress are characterized by ionic- and osmotic-disequilibrium components; eliciting general as well as specific responses and mechanisms of stress-protection [2]. These studies underpinned the importance of *early *responses to the various stresses for the survival of the plants [3]. Much of our current understanding of stress-response mechanisms comes from genome-wide analysis of gene expression, facilitated by the availability of microarrays carrying a comprehensive set of genes.

In chickpea, like in many other under-researched crops, extensive microarray-based studies are not yet possible, because the necessary EST data are not available. For example, no more than 7,580 chickpea ESTs are publicly available at the National Centre of Biotechnology Information (NCBI, ), as compared to at least 1,463,500 ESTs available for *Arabidopsis*. In the absence of such resources, researchers often use less comprehensive approaches as e.g. suppression subtractive hybridisation (SSH) libraries [4], which do not *per se *allow the quantification of expression of differentially expressed genes. SSH results are therefore often used for the generation of macro-arrays for subsequent analysis of gene expression [5]. These authors used this cumbersome approach for the discovery of 101 dehydration-responsive transcripts in chickpea roots.

Open-architecture, whole-genome transcription profiling technologies such as SuperSAGE [6], however, provide a solution to the problem of lacking EST and genomic data. SuperSAGE is an improved version of the Serial Analysis of Gene Expression (SAGE) technique [7]. In the past years, it has demonstrated a high versatility due to its longer tag size (26 bp) [8,9]. In principle, SAGE and all its variants rely on the assumption that a small, defined part of a cDNA, a so-called "tag", characterizes this cDNA, and that counting the number of times a particular tag occurs in the tag population faithfully reflects the abundance of the respective mRNA in the transcriptome. Since 10.000 to 100.000 tags are sequenced in a single experiment, a comprehensive profile of the transcriptome is generated.

Here we report on the stress responses of 80,238 transcripts representing 17,493 unique 26 bp tags (UniTags) from roots of the drought-tolerant chickpea variety ICC588 early after onset of desiccation. We discuss the stress-regulated transcription of genes involved in signal perception and transduction, ROS scavenging and metabolism, osmotic and ionic stress-related pathways, regulation of water and ion homeostasis, as well as several reported effector proteins. To test the reliability of the present results we use microarrays carrying stress-responsive as well as constitutively expressed 26 bp tag sequences. To further confirm the SuperSAGE results with a third method, we use SYBRgreen and commercially available *Taq*Man assays produced from 3'-and 5'-RACE sequences from selected chickpea mRNAs. Finally, we compare our transcription profiles from drought-stressed chickpea roots to results obtained from chickpea root SSH libraries [5] and microarray experiments in *Medicago truncatula *[10], and discuss similarities and differences. This study is the first of a series characterizing stress responses of chickpea on a molecular level as a prerequisite for production of expression markers and microrrays for high-throughput germplasm and expression (e)QTL analysis at the onset of knowledge-based breeding for stress-tolerance in this important protein crop.

## Results

### The combination of high-throughput 454 sequencing with SuperSAGE

Drought libraries are part of a project, which aims at evaluating the transcriptional responses of chickpea upon diverse abiotic stresses, including several other treatments and various tissues (e.g. salt-stressed roots and cold-stressed leaves; data not shown). For the sequencing of all the libraries, a single 454 plate divided into two sections was used, from which a total of 380,000 reads were extracted. After eliminating: i) incomplete reads, ii) twin-ditags, and iii) ditags without complete library-identification DNA linkers, a total of 330,000 26 bp tags were obtained for further analysis. From these, about 50,000 tags were singletons, that were excluded from analysis. Finally, 280,000 tags remained for evaluation (data not shown). Subsequently, sub-datasets were constructed for each experimental situation. For the present study, 82,238 26 bp tags from control and dehydrated roots were analyzed.

### Abundance of UniTags and annotation to public databases

A total of 82,238 26 bp tags from roots of the drought-tolerant variety ICC588 either subjected to 6 h desiccation (53,141) or from well-watered controls (28,897) were sequenced, and represented 17,493 unique transcripts, so called UniTags. Less than 1% percent of these occurred in very high copy numbers (> 5,000 counts.million^-1^), whereas 23% and 75% of the transcripts were present between 100 to 1,000 and less than 100 copies.million^-1^, respectively (Table 1). UniTags from control and stress libraries were deposited in the Gene Expression Omnibus (GEO) public domain under accessions GSM321783 and GSM321790, respectively.

**Table 1:** Features of SuperSAGE libraries from control and drought stressed roots

**Library**	**Control**	**6 h desiccation**	**Total (%)**
Sequenced tags	28,897	53,141	82,238 (100)
Number of unique transcripts (UniTags)	9,110	13,899	17,493 (100)

**Differential gene expression (absolute values)**	(downregulated*)	(up-regulated*)	
R_(ln) _> 1; 2.7-fold differential expression	4,975	2,557	7,532 (43)
R_(ln) _> 2; 8.0-fold differential expression	589	291	880 (5)

**Abundance classes**			
> 5,000 copies.million^-1^	-	-	12 (0.1)
1,000 – 5,000 copies.million^-1^	-	-	186 (1)
100–1,000 copies.million^-1^	-	-	4,160 (24)
2–100 copies.million^-1^	-	-	13,135 (75)

**Annotation of Unitags**			
Match to UniProt entries	2,124	3,165	3,858 (22)
Match to anonymous entries	-	-	5,685 (32)
No match			7,956 (45)

* Ratio (ln) values indicating up- or down-regulation calculated with the 6 h desiccation library as reference.

Annotation of the 17,493 UniTags matched 3,858 (22.0%) to well characterized sequences from the Fabaceae family available in public databases. Of these, 53% matched to sequences from *Medicago truncatula*, 29% to *Cicer arietinum*, 6% to *Pisum sativum*, 3% to *Glycine sp*., 2% to *Medicago sativa*, and 7% to other legume genera. In many cases, TIGR gene index annotations from legumes bridge automatically to characterized Tentative Consensus (TC) sequences mostly from *Arabidopsis*, rice, and maize. Of the remaining 13,635 (78.0%) non-assignable 26 bp tags, 5,685 were significantly homologous to anonymous EST or DNA sequences, whereas 7,950 found no match at all. A summary of the primary data is given in Table 1. Annotation of the 26 bp tags and respective expression ratio values are deposited in the main data matrix [see Additional file 1].

### Annotation of virtual tags generated from chickpea ESTs deposited in public domains

In order to test the validity of the annotation of chickpea 26 pb tags through sequence homology with other legumes, virtual tags generated from chickpea EST sequences deposited in the NCBI data bank were extracted, and their direct annotation was compared with the annotation of longer homologous ESTs from the model legume *M. truncatula*. After retrieving the complete set of chickpea sequences deposited in the NCBI EST database (7,500 sequences), a total of 3,544 different *in silico*-generated 26 bp tags were selected to be directly BLASTed against the nr NCBI (*Fabaceae*) nucleotide database (Table 2). From these, a total of 998 tags revealed high homology hits. After exclusion of anonymous entries, 253 tags were linked to Uniprot entries or to characterized non-protein coding RNAs (Table 3). In parallel, the same 3,544 tags were BLASTed against the plant EST NCBI (*M. truncatula*) and TIGR (*M. truncatula*) databases separately, where 1,143 and 680 sequences, respectively, found at least one high homology hit. Then, the complete target sequences from each BLAST were retrieved and re-blasted against the nr NCBI (*Fabaceae*) database. A total of 632 (NCBI *M. truncatula *ESTs) and 630 (*M. truncatula *TIGR ESTs) sequences, each representing a different 26 bp tag, revealed high homologies with nr NCBI entries. From these, 369 and 213, respectively, were non anonymous (Table 3). The results from the direct BLAST were compared with each of the *M. truncatula *EST-briged homology searches for commonly annotated sequences, either by direct ID matching, protein name, or sequence description. For *M. truncatula *ESTs from NCBI, 9 out of 114 (7.8%) annotations were not congruent with the annotation of the corresponding chickpea *in silico *tag. However, only 4 out of the 9 annotations belonged to Uniprot-linked accessions [Table 3, see also Additional file 2]. For *M*. *truncatula *ESTs from TIGR, 9 out of 86 (10%) common annotations were not congruent. From them, 4 belonged to Uniprot-linked accessions [Table 3, see also Additional file 2]. The present results reveal that in more than 90% of the cases, the assignment of tags to Uniprot-linked accessions is congruent with the annotation of longer ESTs from other legumes. However, databases overloaded with anonymous entries considerably reduce the amount of biologically interpretable data.

**Table 2:** Chickpea EST sequences used for in silico extraction of 26 bp tags

**Chickpea ESTs deposited at NCBI**	7584
**ESTs with more than 30 bp between last CATG and 3'-end**	4754

**Different *in silico *26 bp tags after virtual *Nla*III cutting and elimination of identical sequences **	3544

**Table 3:** Comparison between direct BLASTing of 26 bp chickpea tags and sequence homology search through M. truncatula ESTs

**Process**	***In silico *tags direct BLAST (I)**	**EST-bridged BLAST (II)**	**EST-bridged BLAST (III)**
			
**Screened databases**	NCBI nr *Fabaceae mRNAs*	NCBI *M. truncatula *ESTs	TIGR *M. truncatula *GI ESTs
			
**Number of BLASTed *in silico *chickpea tags**	3544	3544	3544
			
**Total high homology hits**	998	632	630
			
**Non-anonymous hits**	253	369	213
**Common hits with NCBI *Fabaceae *direct BLAST**	-	114	86
			
**Direct ID-correlated common hits**	-	48	37
			
**Protein name-correlated hits**	-	42	23
			
**RNA description-correlated hits**	-	15	17
			
**Non-correlated hits**	-	9	9

### Differential gene expression in response to drought stress of chickpea roots and assignment of 26 bp tags to Gene Ontology (GO:) functional categories

**Figure 1:**
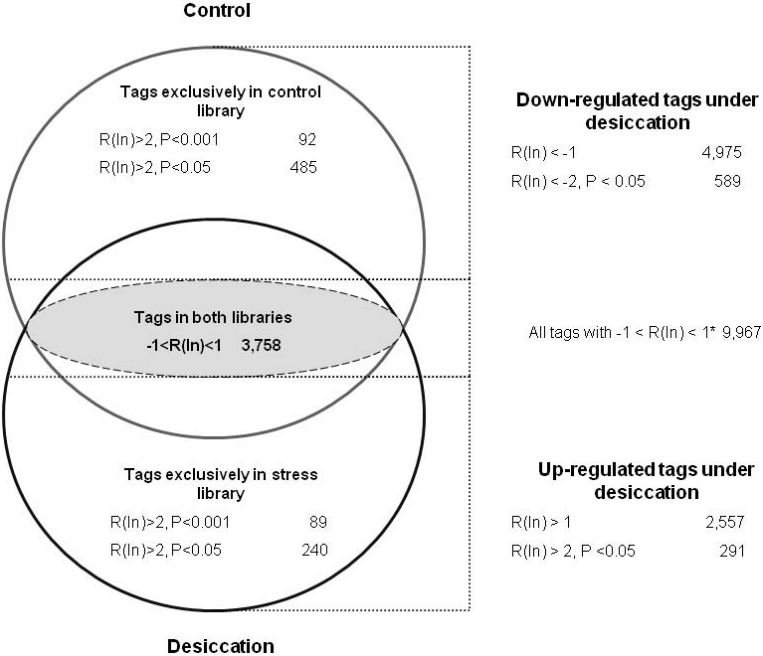
**Venn diagram showing down-regulated, constitutively expressed, and up-regulated chickpea 26 bp tags**. Venn diagram of the quantitative tags classification into down-regulated, constitutively expressed, and up-regulated transcripts in control and drought-stressed chickpea roots. * Tags not differentially expressed, including tags found in either the control or the drought-stress library, respectively (at low statistical significance). Several differentially expressed tags are observed in both libraries.

We calculated the natural logarithm of expression ratios [here denoted as R_(ln)_] of the 26 bp tags from control versus stressed roots as well as significance levels (P) according to Audic and Claverie [11] of up- and down-regulation for each transcript using the software package DiscoverySpace 4.01 (Canada's Michael Smith Genome Sciences Centre). Differences in abundance of tags in control and stressed roots were considered relevant at R_(ln) _> 1 (> 2.7-fold change). At this threshold, 7,532 (43%) tags were significantly differentially expressed in stressed as compared to control roots. Of these, 2,557 were up-, and 4,975 down-regulated. A total of 880 transcripts (5.0%) showed more than 8-fold difference in expression (R_(ln) _> 2.0, P < 0.05). Of these, 291 were up-, and 589 down-regulated under stress. As depicted in the Venn diagram (Figure 1), a considerable number of tags occurred exclusively in either the control tissue or under stress.

The 40 most significantly up- or down-regulated transcripts matching well characterized genes in public databases are listed in Table 4 and Table 5, respectively. However, many transcripts could not be annotated, because they either matched to anonymous ESTs, or did not hit any entry in the database. Therefore, the annotatable transcripts coding for extensin (O65760_CICAR), NADP-dependent isocitrate dehydrogenase I (Q6R6M7_PEA), S-receptor kinase-like protein 1 (Q70I30_LOTJA), chalcone isomerase (Q9SXS9_CICAR), UDP-glucose-pyrophosphorylase (Q8W557_9FABA), beta-glucosidase (Q9FSY8_CICAR), specific tissue protein 1 (Q39449_CICAR), S-adenosyl-L-methionine synthetase (Q9AT56_ELAUM), and cysteine synthase (O65747_CICAR) were the most up-regulated interpretable transcripts under stress though not the most up-regulated in the data set.

**Table 4:** Top 40 drought stress up-regulated annotatable tags

**Tag code**	**Protein**	**R_(ln)_**	**GO: Biological process**	**Uniprot ID**
STCa-19021	Extensin	3,694	Cell wall organization and biogenesis	O65760_CICAR
STCa-7166	NADP-dependent isocitrate dehydrogenase I	3,579	Carbohydrate metabolism	Q6R6M7_PEA
STCa-7800	S-receptor kinase-like protein 1	3,579	Protein amino acid phosphorylation	Q70I30_LOTJA
STCa-10145	Chalcone isomerase	3,517	No associated term	Q9SXS9_CICAR
STCa-8459	UDP-glucose pyrophosphorylase	3,341	Metabolism	Q8W557_9FABA
STCa-228	Beta-glucosidase	3,261	Carbohydrate metabolism	Q9FSY8_CICAR
STCa-20422	Specific tissue protein 1	3,218	No associated term	Q39449_CICAR
STCa-23486	S-adenosyl-L-methionine synthetase	3,127	One-carbon compound metabolism	Q9AT56_ELAUM
STCa-2982	Cysteine synthase, O-acetyl-L-serine (thiol)-lyase	3,079	Metabolism	O65747_CICAR
STCa-22698	Putative adenosine kinase	2,916	Purine ribonucleoside salvage	Q8L5Q4_CICAR
STCa-17627	Putative universal stress protein	2,791	Response to stress	Q700A7_CICAR
STCa-542	Prolyl 4-hydroxylase, alpha subunit-like protein	2,722	Protein metabolism	Q9FKX6_ARATH
STCa-1589	Beta-galactosidase	2,722	Carbohydrate metabolism	O65736_CICAR
STCa-2044	Fiber protein Fb11	2,648	No associated term	Q8GT82_GOSBA
STCa-227	Beta-glucosidase	2,568	Carbohydrate metabolism	Q9FSY8_CICAR
STCa-866	Protein kinase Pti1	2,568	Protein amino acid phosphorylation	Q84P43_SOYBN
STCa-15340	Alfin-1	2,568	Regulation of transcription	Q40359_MEDSA
STCa-16114	Cytosolic acetoacetyl-coenzyme A thiolase	2,568	No associated term	Q5XMB8_TOBAC
STCa-16514	NADH dehydrogenase	2,568	Mitochondrial electron transport	Q9FNN5_ARATH
STCa-5543	Epsilon subunit of mitochondrial F1-ATPase	2,525	ATP synthesis coupled proton transport	Q8L5Q1_CICAR
STCa-8853	Ribosomal protein L10 homolog	2,481	Protein biosynthesis	Q42149_ARATH
STCa-857	Histone H2B	2,386	Response to DNA damage stimulus	Q9M3H6_CICAR
STCa-21625	Serine protease inhibitor-like protein	2,386	No associated term	Q8RV99_ORYSA
STCa-24140	Putative 14-kDa proline-rich protein	2,386	Llipid transport	Q9LEN8_CICAR
STCa-16415	NADPH-cytochrome P450 oxidoreductase	2,36	Electron transport	Q7M275_TOBAC
STCa-923	Ribosomal protein S26	2,28	Protein biosynthesis	Q9SWS9_PEA
STCa-1343	Apyrase-like protein	2,28	No associated term	Q84UE1_MEDTR
STCa-2122	Histone H2A	2,28	Chromosome organization	H2A_CICAR
STCa-6603	Polygalacturonase PG11 precursor	2,28	Carbohydrate metabolism	Q84TM8_MEDSA
STCa-7388	Aldolase	2,28	No associated term	Q945F2_CICAR
STCa-8045	CaM protein	2,28	Calcium related signal transduction	Q7DLT8_CICAR
STCa-14940	TGA-type basic leucine zipper protein TGA2.1	2,28	Regulation of transcription	Q93XA1_PHAVU
STCa-15506	Delta-COP	2,28	Intracellular protein transport	Q9M640_MAIZE
STCa-16257	ABA-responsive protein	2,28	Stress Response/ABA dependent	Q9FMW4_ARATH
STCa-16760	Elongation factor 1-alpha	2,28	Protein biosynthesis	O81921_CICAR
STCa-24349	Gibberellin 2-beta- hydroxylase	2,28	Antibiotic biosynthesis	G2OX_PHACN
STCa-24395	NADPH:isoflavone oxidoreductase	2,28	Regulation of nitrogen utilization	IFR_CICAR
STCa-24453	Tonoplast intrinsic protein	2,28	Transport	Q8L5G0_CICAR
STCa-89	Drought-induced protein	2,162	Response to water stress	Q941N0_9FABA
STCa-1016	Protein phosphatase 1, catalytic beta subunit	2,162	Protein amino acid de-phosphorylation	O65844_MEDSA

**Table 5:** Top 40 drought stress down-regulated annotatable tags

**Tag code**	**Protein**	**R_(ln)_**	**Biological process**	**Uniprot ID**
STCa-1804	Expansin-like protein (fragment)	-3,095	Sexual reproduction	Q7XHJ2_QUERO
STCa-13652	40S ribosomal protein S23	-3,095	Protein biosynthesis	RS23_EUPES
STCa-4802	ADP-glucose pyrophosphorylase precursor	-2,913	Glycogen biosynthesis	Q43819_PEA
STCa-5076	Ribosomal protein L32	-2,913	Protein biosynthesis	Q45NI6_MEDSA
STCa-7347	Putative 3-hydroxybutyryl-CoA dehydrogenase	-2,913	Fatty acid metabolism	Q9LDF5_ARATH
STCa-8227	Histone H3	-2,913	Chromosome organization	H3_ONOVI
STCa-13267	Allene oxide synthase precursor	-2,913	Lipid biosynthesis	Q7X9B4_MEDTR
STCa-17859	Hypothetical protein 275	-2,913	No associated term	Q8GTD8_CICAR
STCa-21081	Vestitone reductase	-2,862	Cellular metabolism	Q40316_MEDSA
STCa-3331	60S ribosomal protein L18	-2,69	Protein biosynthesis	RL18_CICAR
STCa-10792	Calcineurin B-like-interacting protein kinase	-2,69	Signal transduction	Q84XC0_PEA
STCa-12317	Heat shock protein 70-3	-2,69	Response to unfolded protein	Q67BD0_TOBAC
STCa-18274	NADPH-ferrihemoprotein reductase	-2,69	Electron transport	Q43235_VICSA
STCa-19040	DNA-directed RNA polymerase subunit B	-2,69	Transcription	Q70Q06_VICSA
STCa-19432	KI domain interacting kinase 1-like protein	-2,69	Protein amino acid phosphorylation	Q9T058_ARATH
STCa-19785	Reduced vernalization response 1	-2,69	Regulation of transcription	Q8L3W1_ARATH
STCa-19870	Transaldolase	-2,69	Carbohydrate metabolism	O04894_SOLTU
STCa-18410	Cytochrome P450	-2,556	Electron transport	Q9ZRW6_CICAR
STCa-18321	Similar to the auxin-independent growth promoter	-2,491	No associated term	Q9LIN9_ARATH
STCa-1286	Eukaryotic translation initiation factor iso4E	-2,402	Translational initiation	Q7XJB0_LACSA
STCa-3390	Phosphoenolpyruvate carboxylase	-2,402	Carbon utilization	CAPP_PHAVU
STCa-3855	ThiF family protein-like	-2,402	No associated term	Q653N8_ORYSA
STCa-3897	20S proteasome alpha subunit C	-2,402	Ubiquitin-dependent protein catabolism	PSA4_SPIOL
STCa-5074	Pectin methyl-esterase PER precursor	-2,402	Cell wall modification	Q9SC90_MEDTR
STCa-5237	F-box family protein-like	-2,402	No associated term	Q5VR67_ORYSA
STCa-5681	Hydroxyproline-rich glycoprotein	-2,402	Cell wall organization	Q39865_SOYBN
STCa-6267	Transcription factor MYBS3	-2,402	Regulation of transcription	Q8H1D0_ORYSA
STCa-6374	Putative extensin	-2,402	Cell wall organization and biogenesis	Q9FSY9_CICAR
STCa-6426	Protein kinase	-2,402	Protein amino acid phosphorylation	Q9ZRU3_CICAR
STCa-6928	40S ribosomal protein S19	-2,402	Protein biosynthesis	Q9ZRW2_CICAR
STCa-6991	Cytochrome P450	-2,402	Electron transport	Q9XGL7_CICAR
STCa-7688	Narf-like protein	-2.402	Electron transport	Q5VR67_ORYSA
STCa-8832	*Cicer arietinum *mRNA for chalcone synthase	-2.402	Biosynthesis	Q39865_SOYBN
STCa-9049	Translocon-associated subunit alpha precursor	-2.402	No associated term	Q8H1D0_ORYSA
STCa-9308	Aquoporin-like water channel protein (mip1 gene)	-2.402	Transport	Q9FSY9_CICAR
STCa-11376	60S ribosomal protein L10 (QM protein homolog)	-2.402	Protein biosynthesis	Q9ZRU3_CICAR
STCa-11527	Putative Bet v I family protein (bet gene)	-2.402	No associated term	Q93YF9_MEDTR
STCa-12919	14-3-3-like protein	-2.402	Protein domain specific binding	Q9ZRV7_CICAR
STCa-13826	Coatomer alpha subunit-like protein	-2.402	Protein targeting	SSRA_ARATH
STCa-14803	ATP synthase alpha chain, mitochondrial	-2.402	ATP synthesis coupled proton transport	Q8GTE0_CICAR

**Figure 2:**
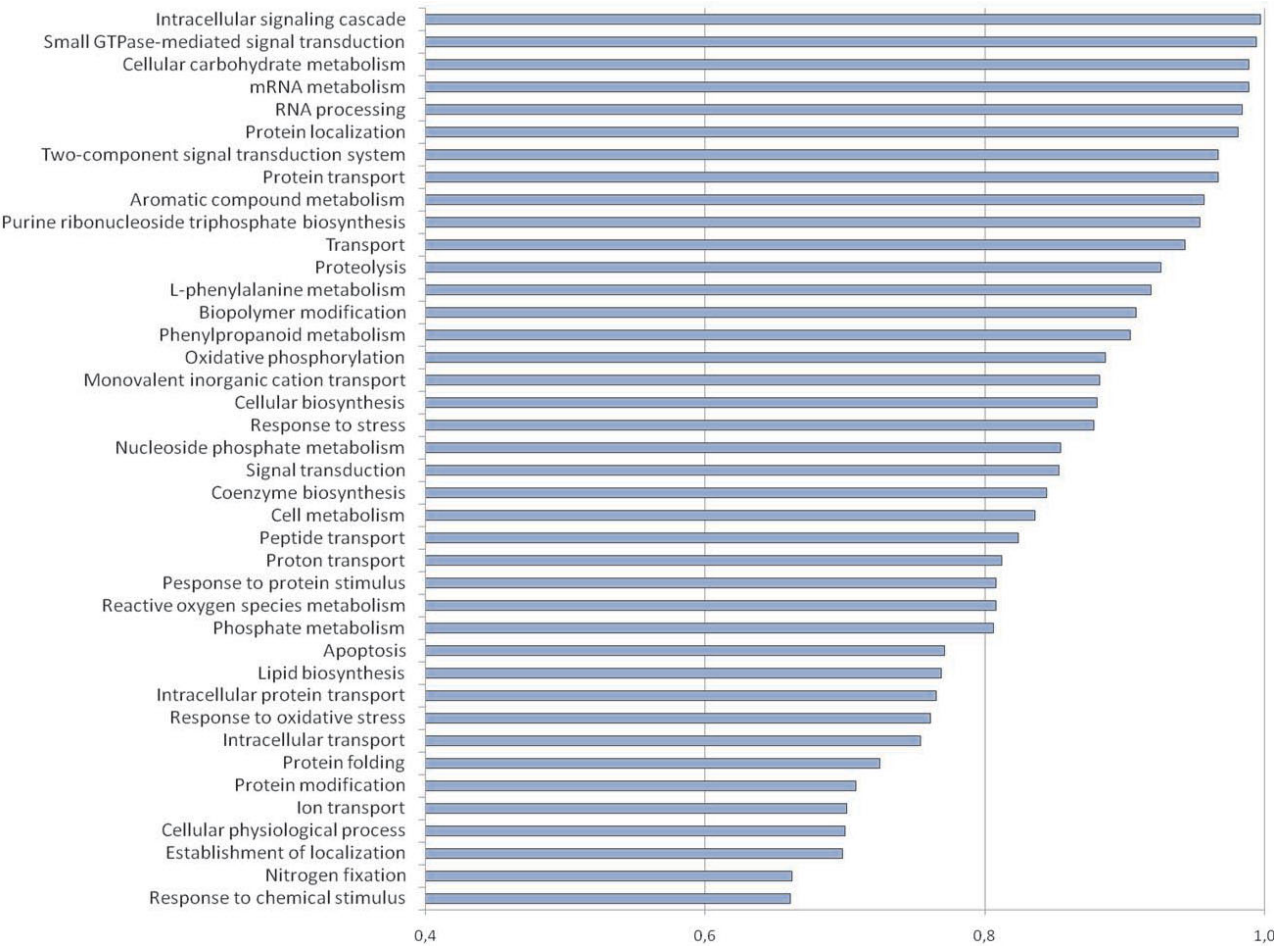
**Drought stress over-represented GO biological processes in chickpea**. Over-represented GO: biological processes as deduced from transcript abundancies (annotated to UniProt entries) in drought-stressed chickpea roots. Representation of GO: terms was calculated by the program ermineJ 2.0. Categories with values above 0.8 are better represented in a given data set.

Correlation of R_(ln) _to defined standard functional gene categories (i.e. biological processes) in the Gene Ontology (GO:) database revealed that the majority of the most up-regulated transcripts are assigned to the GO: biological process "Metabolism" (with the exception of extensin [O65760_CICAR], and S-receptor kinase-like protein [Q70I30_LOTJA] transcripts). As depicted in Figure 2, Stress Perception and Signalling (i.e. Intracellular Signalling Cascades, P = 0.997), Small GTPase-mediated Signal Transduction (P = 0.994), RNA Metabolism (P = 0.989), and Cellular Carbohydrate Metabolism (P = 0.989) were the most represented GO: functional categories in desiccation-stressed roots. Further, over-representation of transcripts involved in Transport (P = 0.943), Proteolysis (P = 0.926), Oxidative Phosphorylation (P = 0.886), and Stress Response (P = 0.878) indicate the mechanisms by which the roots adapt to the stress. Over-representation of transcripts involved in GO: functional category "Oxygen and Reactive Oxygen Species Metabolism" (P = 0.808) suggests that ROS play an important role as side stress, but also as signalling molecules.

**Figure 3:**
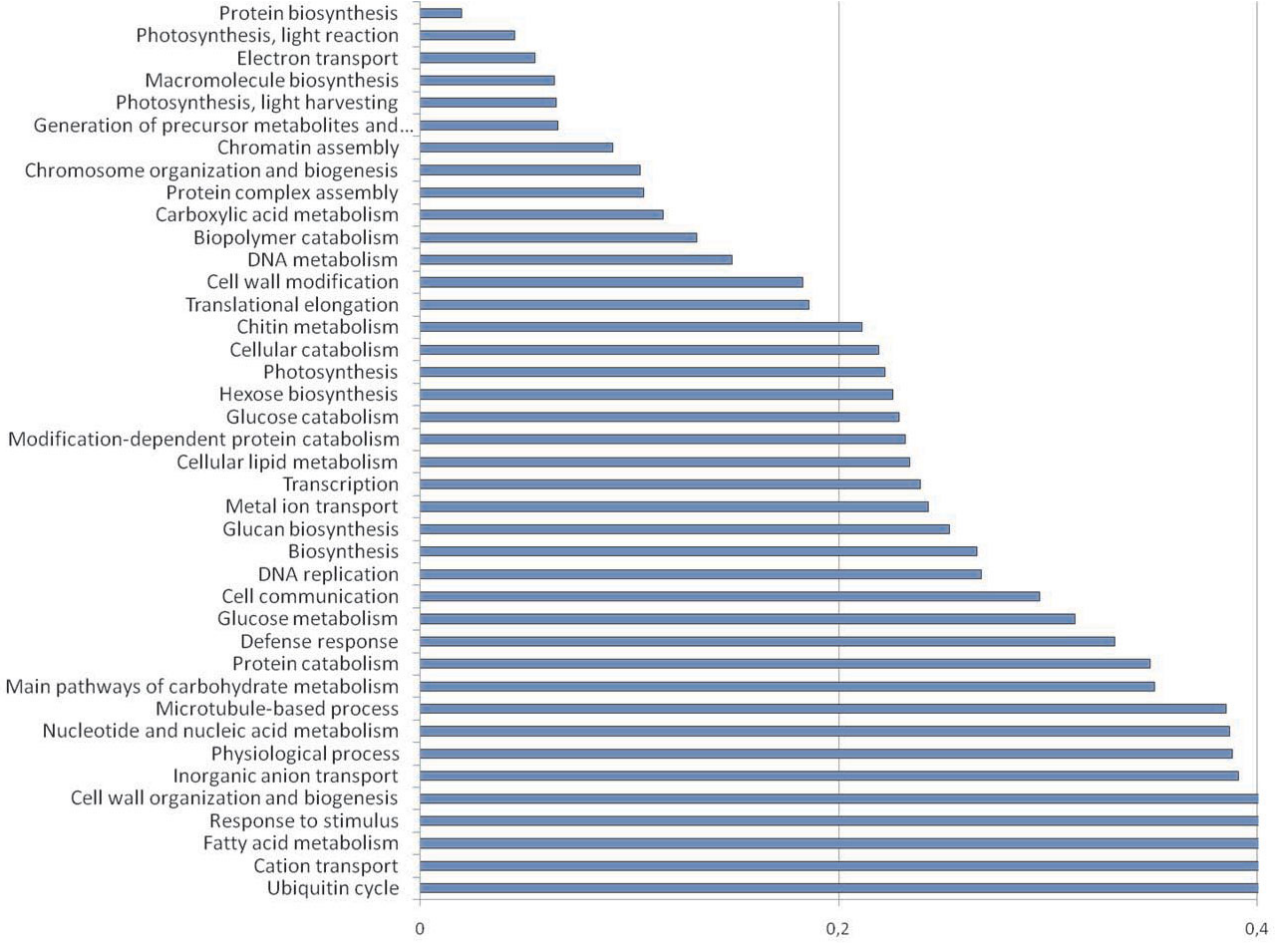
**Drought stress under-represented GO biological processes in chickpea**. Under-represented GO: biological processes as deduced from transcript abundancies (annotated to UniProt entries) in drought-stressed chickpea roots. Representation of GO: terms was calculated by the program ermineJ 2.0. Categories with values below 0.2 are less represented in a given data set.

Tags from transcripts assigned to the GO: biological process "Protein Biosynthesis" (P = 0.020), such as 40S ribosomal proteins S19 (Q9ZRW2) and S23 (RS23), and 60S ribosomal proteins L10 (Q9ZRU3), L18 (RL18), and L32 (Q45N16), all were significantly down-regulated suggesting a repression of *de novo *protein biosynthesis under drought stress in roots. Additionally, other GO: biological processes like Photosynthesis and Light Reaction (P = 0.045), Chromatin Assembly (P = 0.092), Chromosome Organization and Biogenesis (0.105), Biopolymer Metabolism (P = 0.132), DNA Replication (P = 0.268) were also under-represented (Figure 3).

**Figure 4:**
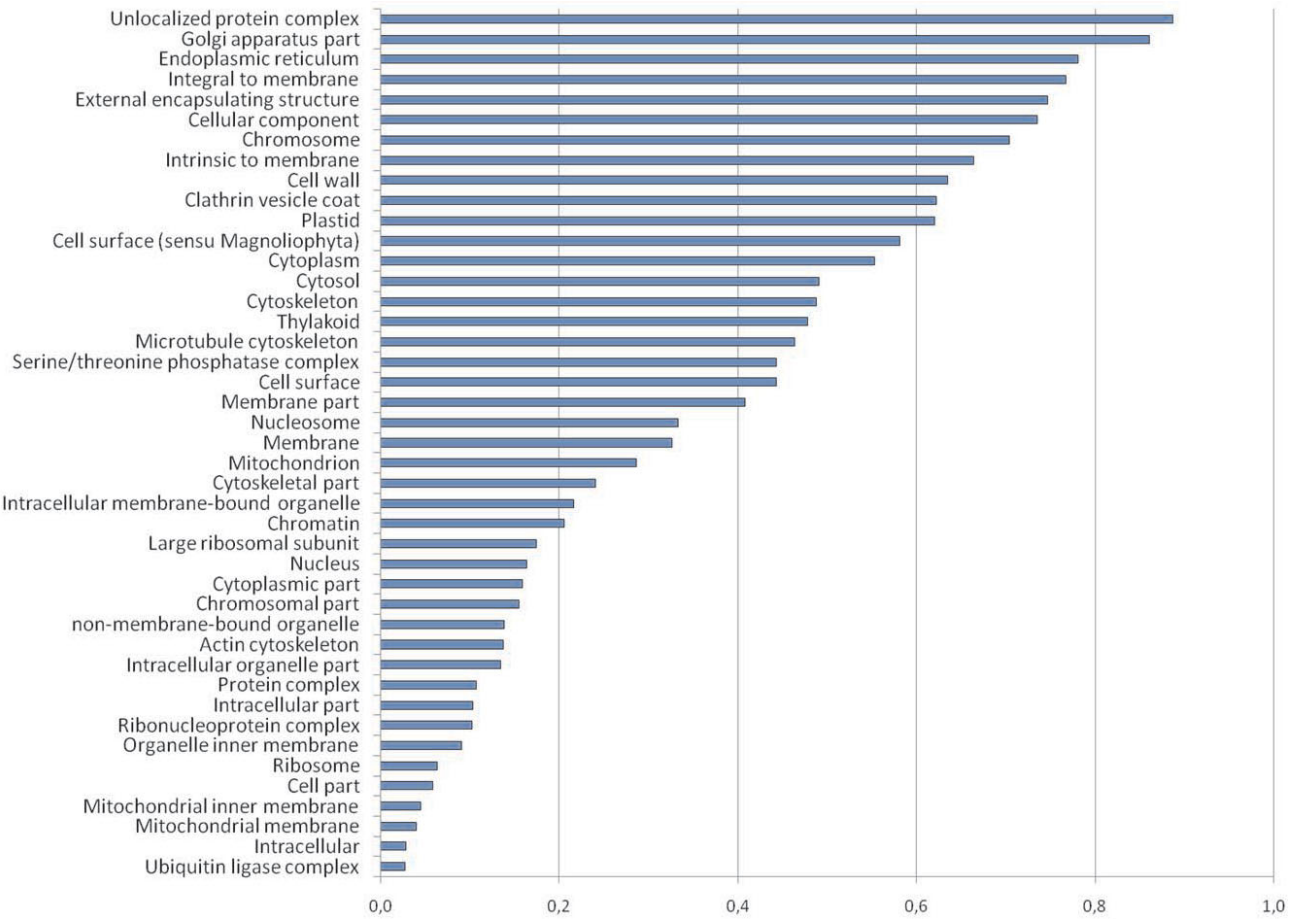
**Drought stress over-represented GO cellular components in chickpea**. GO: cellular components assigned to localization as deduced from transcript abundancies (annotated to UniProt entries) in drought-stressed chickpea roots. Representation of GO: terms was calculated by the program ermineJ 2.0. Categories with values above 0.8 are better represented in a given data set.

Regarding GO: Cellular component categories, "Unlocalized Protein Complexes" were most over-represented under drought stress (P = 0.887), followed by Golgi Apparatus (P = 0.861), Endoplasmic Reticulum (P = 0.781), Membrane Integral Genes (P = 0.767) and External Encapsulating Structure (P = 0.747). GO: Cellular components such as "Ubiquitin Ligase Complex" (P = 0.027), Mitochondrial and Inner Membrane (P = 0.040 and 0.044, respectively), and Ribosome (P = 0.063) were amongst the most under-represented. The transcription of genes coding for proteins of the Serine/Threonine Phosphatase Complex (P = 0.44), Cytoplasm (P = 0.55), Cytosol (P = 0.49), Cytoskeleton (P = 0.48), Thylakoid (P = 0.47), Microtubule Cytoskeleton (P = 0.46), Cell-wall Structure and Modification as well as Cell Surface Protein was constitutive (Figure 4).

Members of a gene family assigned to over- or under-represented GO: categories, respectively, could be up- as well as down-regulated to different extents. For example, whereas UniTagSTCa-6374, annotated to the putative extensin (Q9FSY9_CICAR), was one of the most down-regulated transcripts, UniTag STCa-19021 representing another extensin isoform (O65760_CICAR) was the most Up-regulated tag in our data set. However, both isoforms are grouped in the same GO:category.

### Differential expression of drought stress-related sub-transcriptomes in chickpea roots

**Figure 5:**
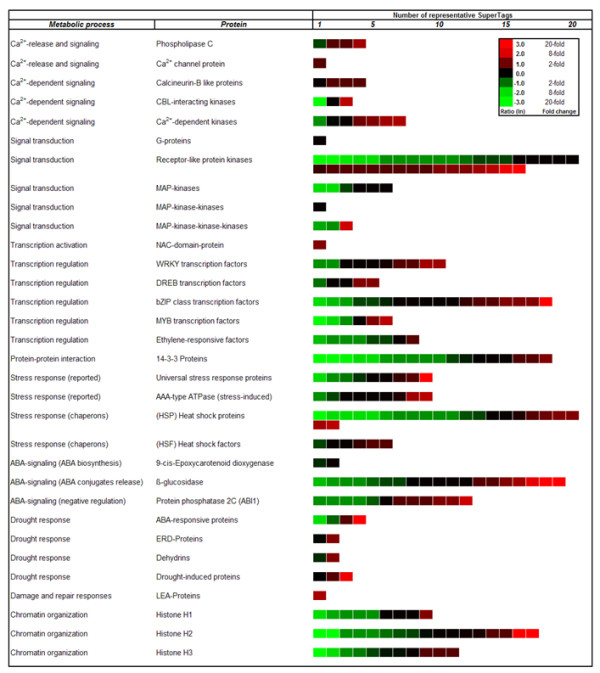
**Heat map profiles of tags representing genes involved in signalling, and response-related processes**. Heat map profiles of tags representing genes involved in Ca^2+^-dependent signalling, general signal transduction, transcription regulation, protein-protein interactions, stress, ABA-dependent signalling, drought-response, damage and repair responses, and chromatin organization.

**Figure 6:**
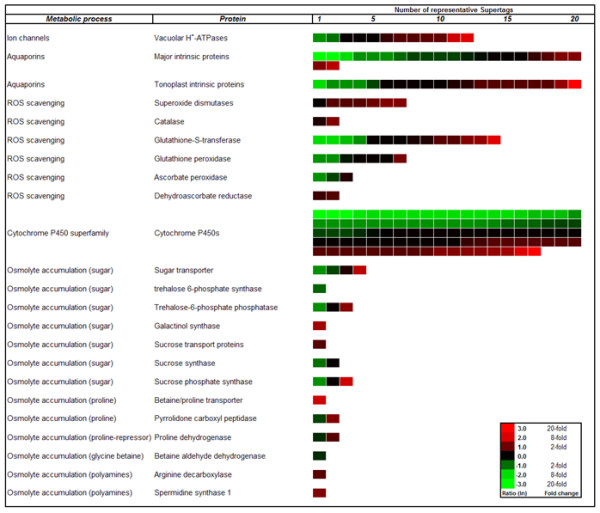
**Heat map profiles of tags representing genes involved in channelling and detoxification-related processes**. Heat map profiles of 26 bp tags representing genes involved in water and ion channelling, ROS detoxification, and compatible osmolyte accumulation, with profiles of 97 26 bp tags annotated to CYP450 genes.

Since the present genome-wide expression analysis revealed a plethora of differentially expressed 26 bp tags with and without match to genes of known function, it is impossible to display or discuss all of them in the frame of this paper. Instead, in Figure 5 and Figure 6 we elaborate in more detail on the expression of genes and gene families belonging to the GO: biological processes "Signal Transduction", "Stress Sensing", "Regulation of Transcription", "Transport", "Post-transcriptional Regulation" and "Pathway Inhibitors", all involved in early responses to stress. In these categories, the gene itself or members of its family have known functions in stress-perception, stress-signalling and stress-responsive regulation of transcription and chromatin structure. Further, we closely look at transcripts encoding proteins involved in Reactive Oxygen Species (ROS) scavenging, and transcripts related to ROS-mediated signal transduction cascades (Figure 6). As examples for the regulation of effector genes down-stream of the signalling cascades, we will detail the differential expression of genes coding for water-channel proteins, so called aquaporins, and of genes coding for proteins involved in osmolyte metabolism (Figure 5, Figure 6).

### Confirmation of SuperSAGE results by qRT-PCR

Our genome-wide expression analysis results were exemplarily confirmed by quantitative real-time (qRT) PCR using *Taq*Man probes XPTm-Ca-22356 (O65741_CICAR, mRNA for putative transmembrane channel protein) and XPTm-Ca-7975 (anonymous drought-induced EST) available from GenXPro GmbH, as well as SYBR Green^® ^assays. Oligonucleotides for SYBR Green assays were deduced from 5'- and 3'-RACE sequences generated with 26 bp tags as primers. The following transcripts were targeted: ST-Ca1921 (O65760_CICAR, extensin), ST-Ca17627 (Q700A7_CICAR, putative universal stress protein), ST-Ca8434 (anonymous drought-induced EST), ST-Ca17859 (AJ515033, *C. arietinum *hypothetical protein [275 gene]), ST-Ca8000 (AJ250836, *C. arietinum *PAL gene), and ST-Ca22717 (AJ487043, *C. arietinum *CYP450). For SYBR Green^® ^as well as *Taq*Man assays, the sequence for either the forward or the reverse PCR primer was derived from the 26 bp tag, and the complementary primers from 3'- or 5'-RACE sequences, respectively.

**Figure 7:**
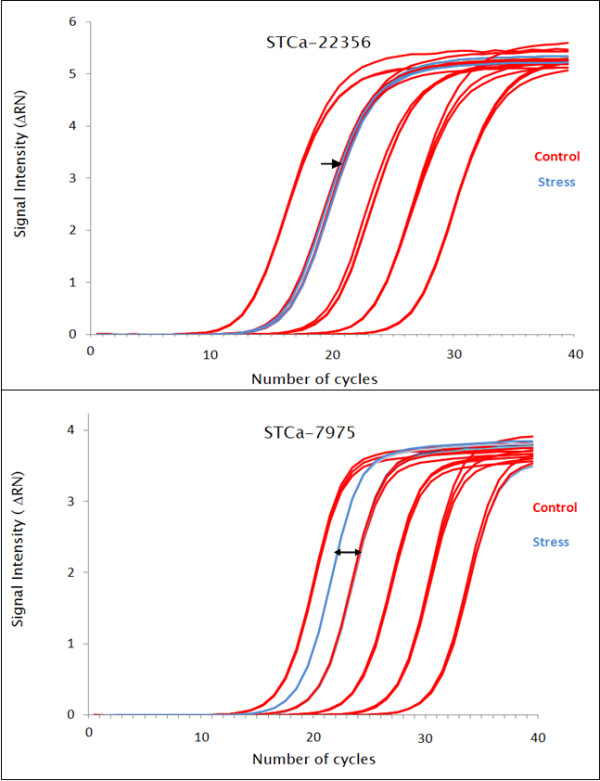
**qRT-PCR confirmation of SuperSAGE results**. TaqMan™ RT-PCR confirmation of SuperSAGE data by relative curve quantification using primers and TaqMan™ probes designed from 3'- and 5'-RACE sequences, respectively, that are derived from corresponding 26 bp tags. A) TaqMan™ assay with tag primer XPTm-Ca-22356 (derived from a 3'-RACE product; the original tag represents O65741_CICAR, the mRNA for a putative trans-membrane channel protein). Result: no difference between control and desiccated roots, i.e. constitutive expression (black arrow). B) TaqMan™ assay with tag primer XPTm-Ca-7975 (derived from a 3'-RACE product; the original tag represents an anonymous drought-induced EST). Result: earlier C_T _for the cDNA from desiccated roots (double headed arrow). Control (red), drought-stressed roots (blue).

Confirming the SuperSAGE expression levels, amplifications from SYBR Green^® ^assay ST-Ca2271 and *Taq*Man probe XPTm-Ca22356 revealed constitutive levels of expression (ΔΔCt < 0.5) (Figure 7). Amplifications for SYBR Green^® ^assays with ST-Ca1921, ST-Ca17627, ST-Ca8434 as well as *Taq*Man probe XPTm-Ca-7975 revealed up-regulation of the respective transcripts under stress (ΔΔCt > 0.5), as already indicated by the differential expression analysis. Stress-induced down-regulation of 26 bp tags was corroborated by SYBR Green^® ^assays for ST-Ca17859 and ST-Ca8000 (ΔΔCt < -0.5). However, for ST-Ca8000, amplification profiles as well as post-qRT-PCR amplicon melting curves suggested partially unspecific priming. Again, in agreement with the SuperSAGE results, the transcript chosen as invariable control indeed displayed almost completely similar expression in control and stressed roots. These experiments confirm our present results and suggest 26 bp tags as reliable sequence information source for other expression profiling techniques.

### Profiles confirmation via microarray hybridization of spotted SuperSAGE-derived oligos

To characterize the chickpea transcriptome under drought stress, sequence information from SuperSAGE profiles was used to design an Agilent 16 K microarray, onto which 3,000 selected 26 pb tags were spotted for a comparison of both profiling techniques. On the microarray, the majority of the oligonucleotides with original tag sequences were spotted twice (twin-replicas). Additionally, oligonucleotides with different mismatch numbers from each original tag as well as a small sub-set of longer RACE-derived sequences were also included. After statistical treatment of the different internal twin-replicas, normalization, and mismatch background correction, reproducible signals from different hybridization rounds (i.e. RNA replicas, and dye-swapped samples) were selected for cluster analyses together with expression ratios of the 26 bp tags. A total of 79.05% probes on the micro-array shared the same tendency of expression with the respective tag when hybridized with cRNAs prepared from drought-treated roots from ICC588. From a total of 1,056 spots showing congruent results among different replicas and dye-swap experiments, 425 and 417 were up- and down-regulated in both techniques, respectively, whereas 214 spots showed in-congruent results (Table 6). UniTag expression ratios from both techniques are deposited in the main data matrix [see Additional file 1].

**Table 6:** Shared and contrasting tendencies between SuperSAGE and microarray profiles for transcripts from drought-stressed chickpea roots

**Stress/Organ**	**Drought/Roots**	Salt/Roots*
Total selected spots (tags)	1,056	739
Shared up-regulation	425	349
Shared down-regulation	417	233
Contrasting tendency	214	157

*Regulation tendencies for salt-stressed roots in both techniques are included for comparative purposes

### Comparison of different transcript profiling techniques: SuperSAGE versus SSH macroarrays and microarrays

**Figure 8:**
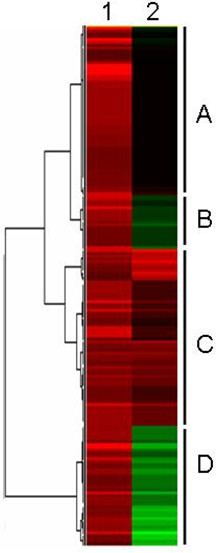
**Comparison of transcription profiles from SSH-derived macro-arrays and SuperSAGE**. Comparison of transcription profiles from SSH-derived macro-arrays (lane **1**, Boominathan et al. 2005) and SuperSAGE, respectively (lane 2). RNA was harvested 5 h (1) or 6 h [81], respectively, after onset of stress. The two original data sets were linked through their UniProt IDs representing each cDNA and 26 bp Tag, respectively. A total of 186 26 bp tags were compared. Transcripts are clustered in groups, that follow similar expression patterns. Four main sections (detailed informations in Table 7) can be distinguished: ***Section A: ***Upregulated transcripts on the macroarray versus constitutive 26 bp tags. ***Section B, D: ***Non-corresponding macroarray and SuperSAGE transcript profiles. ***Section C: ***Upregulated transcripts on the macroarray and upregulated 26 bp tags.

We tested, whether our genome-wide transcription patterns are typical for roots of chickpea and other legumes, even if the profiles were obtained with other methods. Therefore we compared the present results to two studies addressing similar questions. In chickpea, Boominathan and co-workers [5] investigated whether pre-exposure to a dehydration shock improved adaptive responses of the roots during subsequent dehydration treatment. These authors identified 101 dehydration-inducible transcripts by repetitive rounds of cDNA subtraction, differential DNA-array hybridization, and Northern-blot analysis. Additionally, responses to exogenously applied abscisic acid (ABA) were also monitored. Since one analyzed time point was set 5 h after onset of drought, the results of this experiment should be at least partially comparable to our study in which RNA was isolated from roots 6 h after onset of drought stress. However, the results were not 100% congruent. It is important to note, that micro- or macro-arrays do not reliably differentiate between different transcript isoforms from gene families. Hybridization signals may integrate the hybridization intensities over all closely related transcripts, whereas SuperSAGE generates absolute numbers for each transcript variant (isoform). Due to the much deeper coverage of the transcriptome by this technique, almost all differentially expressed transcripts represented in the SSH libraries have at least one, but usually more counterparts in our 26 bp tags libraries. A cluster analysis of transcription profiles obtained by macroarray hybridization and the differential expression of 26 bp tags from drought-stressed roots 5 h or 6 h after onset of the stress, respectively, is shown in Figure 8.

Similarly up-regulated (category C in Figure 8) and differentially regulated transcripts (i.e. down-regulated in the SuperSAGE experiment, but up-regulated on the macroarray, section D in Figure 8) under both conditions are listed in Table 7.

**Table 7:** Comparison between SuperSAGE expression profiles and macroarray-generated data

**Section**	**Annotation**	**Uniprot ACC**
**A**	ADP-glucose pyrophosphorylase precursor	Q43819
	ADP-glucose pyrophosphorylase small subunit CagpS1	Q9AT06
	ADP-ribosylation factor 1-like protein	Q70XK1
	ADP-ribosylation factor-like protein	Q9LFJ7
	AP2/EREBP transcription factor ERF-1	Q5U8L5
	ATP-dependent Clp protease ATP-binding subunit clpC homolog	CLPC
	Chaperonin 21 precursor	Q9M5A8
	Cytochrome P450	Q9XGL7
	Cytochrome P450 73 cinnamic acid 4-hydroxylase	TCMO
	Cytosolic chaperonin, delta-subunit	Q9ZRX1
	Dehydration-responsive element binding protein	Q8GZF2
	Dehydrin-cognate	Q43430
	DREB-like protein	Q75UJ6
	(RING zinc finger protein, putative, expressed)	Q75I59
	Fiber protein Fb22 (Fragment)	Q7Y244
	Fiber protein Fb27	Q6UA10
	Fiber protein Fb4 (Fragment)	Q6UA21
	Glyceraldehyde 3-phosphate dehydrogenase, cytosolic	O81924
	Histone H2A	H2A2
	Kinesin (centromere protein)-like heavy chain-like protein	Q9LHL9
	Lipoxygenase	Q93YA9
	Metallothionein-like protein 1; MT-1	MT1
	PGM; Glucose phosphomutase	PGMC
	Polygalacturonase inhibiting protein	Q6V406
	Protein kinase	Q41619
	Protein phosphatase-2C	O82468
	RING/C3HC4/PHD zinc finger-like protein	Q84KA9
	Root-specific metal transporter	Q84LR1
	S-adenosylmethionine synthase	Q6J9X6
	S-adenosylmethionine synthetase	Q9FUZ1
	Similarity to zinc metalloproteinase	Q9FKI1
	Translation initiation factor 5A	Q6PQ38
	Trehalose-6-phosphate phosphatase	O64897
	Zinc finger (C3HC4-type RING finger)protein-like	Q67WE0
	Zinc finger (CCCH-type) protein-like	Q657B3
	Zinc finger protein 5, ZFP5	Q8LCZ7
	Zinc finger-like	Q6K719

**B, D**	ADP-glucose pyrophosphorylase precursor	Q43819
	ADP-glucose pyrophosphorylase small subunit CagpS1	Q9AT06
	AP2/EREBP transcription factor ERF-2	Q5U8L6
	ATP-dependent Clp protease ATP-binding subunit clpC homolog	CLPC
	Beta-amylase; 1,4-alpha-D-glucan maltohydrolase	AMYB
	Central motor kinesin 1	Q6WJ05
	Cinnamoyl CoA reductase	Q7Y0H8
	Cytochrome P450	Q9XGL7
	Cytochrome P450 73 cinnamic acid 4-hydroxylase	TCMO
	Eukaryotic translation initiation factor 5A isoform II	Q71F50
	Eukaryotic translation initiation factor 5A-2; eIF-5A-2	IF5A2
	Fiber protein Fb19 (Fragment)	Q7X9S1
	Glyceraldehyde 3-phosphate dehydrogenase, cytosolic	O81924
	Glyceraldehyde-3-phosphate-dehydrogenase	Q53I52
	Histone H2A.6	H2A6
	Lipoxygenase (EC 1.13.11.12)	Q9LEA9
	Metallothionein-like protein 1; MT-1	MT1
	Metallothionein-like protein 2; MT-2	MT2
	Nine-cis-epoxycarotenoid dioxygenase1	Q8LP17
	Nodule-enhanced protein phosphatase type 2C	Q9ZPL9
	P-type H+-ATPase	Q41647
	PGM; Glucose phosphomutase	PGMC
	Plasma membrane H+-ATPase	Q7Y066
	Prolyl 4-hydroxylase alpha subunit-like protein	Q8LAN3
	Protein kinase	Q41619
	Protein phosphatase 2C	Q8S8Z1
	Protein phosphatase 2C-like protein (AT4g31860/F11C18)	Q9SZ53
	Protein phosphatase-2C	O82469
	Putative metallophosphatase	Q8VXF5
	S-adenosylmethionine synthetase	Q9FUZ1
	Translation initiation factor 5A	Q6PQ38
	Ubiquitin conjugating protein	Q9M4R0
	Vacuolar assembly protein VPS41 homolog	VPS41
	Zinc finger (CCCH-type) protein-like	Q657B3
	Zinc finger protein-like	Q5Z8K9
	Zinc finger protein-like	Q69QZ4
	Zinc finger protein-like	Q6K8E9

**C**	ADP-glucose pyrophosphorylase precursor	Q43819
	ADP-ribosylation factor 1-like protein	Q70XK1
	Apyrase-like protein	Q84UE1
	Chloroplast chaperonin 21	Q6B4V4
	Cysteine proteinase	O81930
	Cytochrome P450	Q9XGL7
	Cytochrome P450 73 cinnamic acid 4-hydroxylase	TCMO
	Dehydration responsive element binding protein	Q7Y0Y9
	Dehydrin-like protein	Q945Q7
	DNA binding zinc finger protein; Pspzf	Q9ZWJ0
	ERD15 protein	Q39096
	Fiber protein Fb11	Q8GT82
	Fiber protein Fb19 (fragment)	Q6T7D1
	Fiber protein Fb2	Q8GT87
	Glyceraldehyde 3-phosphate dehydrogenase, cytosolic	O81924
	Histone H2A	H2A
	Kinesin-like protein; 73641–79546	Q9CAC9
	Late embryogenesis-like protein (Fragment) (LEA-Prot)	O81366
	Lipoxygenase (EC 1.13.11.12)	O04919
	Lipoxygenase (EC 1.13.11.12)	Q93YI8
	Metalloendopeptidase	Q40983
	Metallothionein-like protein 1; MT-1	MT1
	Metallothionein-like protein 2; MT-2	MT2
	Nonspecific lipid-transfer protein precursor; LTP	NLTP
	P-type H^+^-ATPase	Q9SAW3
	P-type H^+^-ATPase	Q9AR52
	Prolyl 4-hydroxylase, alpha subunit-like protein	Q9FKX6
	Protein phosphatase 2C	O81709
	Putative imbibition protein	Q9M442
	Root-specific metal transporter	Q84LR1
	Rubisco activase	Q8GTY4
	RuBisCO large subunit-binding protein subunit alpha	RUBA
	Thiolprotease	Q41064
	Transcription factor DRE-binding factor 2	Q6IVL3
	Translation initiation factor 5A	Q6PQ38
	Trehalose-6-phosphate phosphatase	O64897
	Ubiquitin conjugating enzyme	O65733
	Ubiquitin conjugating enzyme	Q43780
	Ubiquitin conjugating protein	Q9M4R0
	UDP-glucose pyrophosphorylase	Q8W557
	Vignain precursor (EC 3.4.22)	CYSEP
	Vignain precursor; cysteine proteinase EP-C1	CYSEP

Comparison between SuperSAGE expression profiles and macroarray-generated data (Boominathan and co-authors [5]). Three main categories from the cluster analysis in **Figure 8 **are detailed below.

***Section A: ***Constitutive tags versus upregulated transcripts on the macroarray.

***Section B, D: ***Non-corresponding SuperSAGE and macroarray transcript profiles.

***Section C: ***Up-regulated tags and up-regulated transcripts on the macroarray.

**Figure 9:**
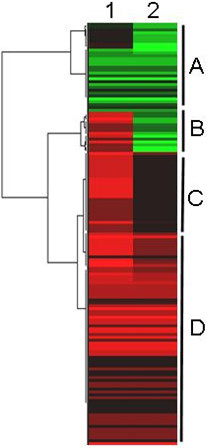
**Comparison of transcription profiles from *Medicago *dessicated young radicles (16k-microarray) with SuperSAGE chickpea profiles**. Comparison of transcription profiles from dessicated young radicles using the *Medicago *16k-microarray (lane 1, Buitink et al. 2006) with SuperSAGE data (lane 2) from drought-stressed chickpea roots. A total of 147 26 bp tags could be linked through the UniProt data base and be used for comparison. Transcripts are clustered in groups, that follow similar expression patterns. Four main categories (detailed informations in Table 8) can be distinguished: ***Section A: ***Down-regulated transcripts on the microarray and down-regulated 26 bp tags. ***Section B: ***Contrasting microarray expression and SuperSAGE profiles (up- vs. down-regulated). ***Section C: ***Contrasting microarray and SuperSAGE profiles (up-regulated vs. constitutive). ***Section D: ***Up-regulated or constitutive 16K-microarray and SuperSAGE transcripts, respectivelyTaqMan™ RT-PCR confirmation of SuperSAGE.

Investigating regulatory and protective mechanisms leading to desiccation tolerance (DT) in *Medicago truncatula *seeds, Buitink and co-workers [10] published another, at least partially comparable study. These authors used the 16 k *Medicago *microarray to monitor changes in the transcriptome of desiccation-sensitive 3-mm-long radicles at different time points during incubation in a polyethylene glycol (PEG) solution mimicking the effects of desiccation. These experiments identified several specific expression profiles at different time scales. A cluster analysis comparing the results from desiccation-stressed *Medicago *radicles and drought-stressed chickpea roots is depicted in Figure 9. Transcripts up-regulated in both species (category D), regulated in opposite directions in the two species (section B), and transcripts down-regulated in chickpea and *Medicago *as a reaction to stress (section A) are listed in Table 8.

**Table 8:** Comparison between SuperSAGE expression profiles and 16 K-microarray-generated expression data

**Section**	**Annotation**	**Uniprot ACC**
**A**	9/13 hydroperoxide lyase	Q7X9B3
	Adenosine 5'-phosphosulfate reductase	Q8W1A1
	Alkaline alpha galactosidase I (Fragment)	Q84NI7
	At1g68060/T23K23	Q8L7S4
	AT3g29575/MWE13	Q94F39
	AT4g18030/T6K21	Q94EJ6
	AT5g03040/F15A17	Q93ZH7
	Auxin-induced beta-glucosidase	Q7XJH8
	Calcineurin B-like-interacting protein kinase	Q84XC0
	ERD3 protein	Q94II3
	Expansin-like protein (Fragment)	Q7XHJ2
	Gb| AAD25781.1	Q9FK34
	General negative transcription regulator-like	Q9LSS9
	Glucan endo-1,3-beta-d-glucosidase precursor	Q9ZP12
	Glucosyltransferase-13 (Fragment)	Q8S996
	Leucine-rich repeat resistance protein-like protein	Q93X72
	Nine-cis-epoxycarotenoid dioxygenase1	Q8LP17
	Photosystem I psaH protein	Q7XA96
	Plasma membrane intrinsic polypeptide	Q9SMK5
	PS60 protein precursor	Q40473
	Putative extracellular dermal glycoprotein	Q9FSZ9
	Putative wound-induced protein	Q9SBR5
	Pyruvate decarboxylase 1 (EC 4.1.1.1)	Q84V95
	Ubiquitin	Q39257

**B**	Basic blue copper protein	Q9ZRV5
	BZIP transcription factor ATB2	Q8L5W2
	Cationic peroxidase	Q9FT05
	CjMDR1	Q94IH6
	Extensin	O65760
	Putative ripening related protein	Q8L6V6
	Putative senescence-associated protein	Q9AVI1
	S-adenosyl-L-methionine	Q84KK6

**C**	Basic blue copper protein	Q9ZRV5
	BZIP transcription factor ATB2	Q8L5W2
	Cationic peroxidase	Q9FT05
	Extensin	O65760
	Hydroxycinnamoyl transferase	Q8GSM7
	Putative ripening related protein	Q8L6V6
	Putative senescence-associated protein	Q9AVI1
	Putative UDP-glycose	Q9M3H8
	Root-specific metal transporter	Q84LR1
	Selenium binding protein	Q93WS1

**D**	1-deoxy-D-xylulose 5-phosphate synthase 1 prec.	Q8L693
	Asparagine synthetase	O24483
	Aspartic proteinase	Q9SXU0
	Aspartic proteinase 2	Q948P0
	AT5g17550/K10A8	Q94EI3
	AT5g64840/MXK3	Q93ZN6
	ATP citrate lyase b-subunit	Q93YH3
	Auxin-induced beta-glucosidase	Q7XJH8
	CjMDR1	Q94IH6
	Ferritin	Q9ZP90
	Gb| AAD25584.1	Q9FJL6
	Glutathione S-transferase GST 11 (EC 2.5.1.18)	Q9FQE7
	Leucine-rich repeat resistance protein-like protein	Q93X72
	LHY protein	Q8L5P7
	Myo-inositol-1-phosphate synthase	Q94C02
	Phosphomevalonate kinase	Q944G1
	Plasma membrane intrinsic polypeptide	Q9SMK5
	Putative extracellular dermal glycoprotein	Q9FSZ9
	RING/C3HC4/PHD zinc finger-like protein	Q84KA9
	1-aminocyclopropane-1-carboxylic acid oxidase	Q84L58
	3-hydroxy-3-methylglutaryl coenzyme A	Q8W2E3
	68 kDa protein	Q9M3Y6
	*Arabidopsis thaliana *TAC clone:K16E1	Q9FH68
	AT3g48690/T8P19	Q8VZG3
	Avr9 elicitor response protein	Q9ZS49
	Basic blue copper protein	Q9ZRV5
	BZIP transcription factor ATB2	Q8L5W2
	Cationic peroxidase	Q9FT05
	Dehydration responsive element binding protein	Q7Y0Y9
	Dehydrin-like protein	Q945Q7
	Drought responsive element binding protein	Q5RM57
	Expansin	Q8GZD3
	Extensin	O65760
	F14J16.29	Q9LG09
	F20N2.11	Q9LFZ9
	F3F9.21	Q9M9E5
	HDA2 (Fragment)	Q8LRK7
	Hydroxycinnamoyl transferase	Q8GSM7
	Hypothetical protein	Q9LEN5
	Importin beta	Q9FJD4
	Ntdin	Q9MBD6
	Polygalacturonase-like protein	Q9LRY8
	Polygalacturonase-like protein; At5g41870	Q9FJ27
	Protein phpsphatase 2C (PP2C) (EC 3.1.3.16)	Q9M3V1
	Putative imbibition protein	Q9M442
	Putative Pi starvation-induced protein	O65757
	Putative quinone oxidoreductase	Q8L5Q7
	Putative ripening related protein	Q8L6V6
	Putative senescence-associated protein	Q9AVI1
	Putative sucrose-H+ symporter	Q84N01
	Putative UDP-glycose	Q9M3H8
	Root-specific metal transporter	Q84LR1
	S-adenosyl-L-methionine	Q84KK6
	Selenium binding protein	Q93WS1

Comparison between SuperSAGE expression profiles and 16K-microarray-generated expression data (Buitink and co-authors [10]). Four main categories from the cluster analysis in **Figure 9 **are detailed below.

***Section A: ***Down-regulated tags and down-regulated transcripts on the microarray.

***Section B: ***Contrasting SuperSAGE and microarray expression profiles (down- versus up-regulated)

***Section C: ***Contrasting SuperSAGE and microarray expression profiles (up-regulated vs. constitutive)

***Section D: ***Up-regulated or constitutive tags and 16K-microarray transcripts, respectively

### Differential expression of natural antisense transcripts (NATs)

As expected from an open-architecture technique, tags synthesized on coding strands of template cDNAs and, in addition, tags originating from the opposite (anti-sense) strands were discovered. Here, at least 170 tags matching to the reverse sequences of ESTs in the public databases were detected. These tags could represent potential natural antisense transcripts (NATs). In *Lotus japonicus*, SAGE tags representing NATs were induced during nodulation [12], and in *Arabidopsis *endogenous siRNAs derived from a pair of natural *cis-*antisense transcripts regulate salt tolerance [13]. Thus, it is possible that NATs are also involved in stress tolerance in chickpea. However, Galante and co-authors [14] demonstrated that a considerable portion of such NATs found in the databases are artefacts. In drought-stressed chickpea roots, we found – *inter alias *– up- and down-regulation of NATs for transcripts of several members of the aquaporin gene family and differential expression of NATs for phosphatase transcripts. However, since we did not exclude genomic priming by DNAse digestion of the RNA prior to cDNA synthesis, confirmation of the identity of these potential NATs will be subject to further studies.

## Discussion

We analysed the expression of approximately 80,000 transcripts from un-stressed control and drought-stressed chickpea roots, respectively. It was previously estimated that the total number of average-sized transcripts per cell in higher plants ranges from 100.000 to 500.000 [15]. Thus, 80.000 26 bp tags provide ~1-fold coverage for transcripts present at a minimum of 6,2 copies per cell. Therefore our study, which detected > 17.000 unique transcripts, is not comprehensive. However, even for the model legume *Medicago truncatula *only 36.000 unique ESTs/TCs are deposited in public databases. Therefore, our single study demonstrates that SuperSAGE is suited to overcome the problem of lacking resources in non-model organisms and under-researched crops.

Since, as we have shown, 75% of transcripts are present in less than 100 copies million^-1^, and a small portion of transcripts is represented by more than 1000 copies million^-1^, we probably missed a number of transcripts with less than 10–14 copies per cell. These may include transcripts for highly interesting pleiotropic proteins such as transcription factors possibly present at only 0,001 copies per cell [16]. Additionally, sampling larger organ sections with millions of cells obscures the specific transcript profiles of component cells. As a consequence, transcripts found in high copy numbers in just a few specialized cells are under-represented. As elegantly demonstrated for maize [17] and *Arabidopsis *[18], there are strong differences in transcription profiles between adjacent tissues. Therefore, the detection of rare transcripts should be improved by increasing the number of sequenced transcripts (as can be done with next-generation sequencing platforms such as Illumina's Solexa or ABI's SOLiD) in combination with a more selective sampling technique such as laser-capture microdissection [19].

Also, annotation of 55% of the 26 bp tags to the databases was straightforward. Considering that roughly 1,300 chickpea EST sequences are publicly available (at the time of annotation), most tags matched to sequences from the related model legume *Medicago truncatula *rather than to chickpea ESTs. On the other hand, a large number of significant hits represented fully uncharacterized database accessions, a fact that handicaps functional interpretation of the present chickpea transcription profiles more than the size of the 26 bp tags.

Additionally, a relevant problem arose from the interpretation of the assignment of different distinct 26bp tags to single UniGenes or tentative consensus sequences (TCs;[20]). We decided to classify such tags as isoforms of transcripts from members of gene families rather than assigning them to a particular gene, even if the expression patterns suggested functional homology. In addition, we could not differentiate whether these tags with similar annotation came from alternatively spliced transcripts of the same gene. Since in *Arabidopsis *and rice at least 21% of genes produce alternatively spliced transcripts [21], evidence beyond sequence homology of the SuperSAGE tags (i.e. 3'- and 5'-RACE sequences) is needed to decide between these two possibilities.

### Differential expression of members of large gene families

One major advantage of the differential expression of 26bp tags as compared to macro- and micro-arrays is the very good differentiation between transcripts from different members of large genes families, which comprise the majority of all genes. Gene family members have more or less conserved sequences, and similar or different functions. For example, the cytochrome P450 (CYP) gene superfamily comprises more than 272 members grouped in 44 families in *Arabidopsis thaliana*, and more than 458 members in rice [22]. In the present study, 97 26bp tags revealed high-homology hits with members of the CYP450 superfamily, from which 33 could be assigned to their respective family through BLASTing against the *Arabidopsis*-CYP database (, data not shown). The involvement of CYP superfamily members in numerous catalytic reactions on a spectrum of substrates suggests that the transcription profiles of its members reflect this diversity. In accordance with the expectation, 43 (44.3%) of the 97 CYP-annotated 26bp tags changed their expression profiles at least 2.7-fold with R_(ln) _(absolute value) > 1.0, whereas 47 (55.7%) showed only slight differences or constitutive levels. Among the 26bp tags assigned to drought stress-related CYPs, hits to CYP707A family (STCa-23852) which harbours ABA 8'-hydroxylases (key enzymes in ABA-catabolism;[23]), revealed a very slight up-regulation (almost constitutive levels), indicating that the turnover of ABA is already active under our experimental condition and time-points. UniTag STCa-18410, assigned to a CYP81F4 member, stands out for its extreme down-regulation (15-fold). Although its function is not known, CYP81F4 members generally change their expression profiles dynamically after induction of water stress [24]. Considering the differential expression and diverse functions of individual members of gene families, whole-genome transcription profiling is only useful, if it differentiates between the different genes and their transcript isoforms in such families. Faithful discrimination between, and individual quantification of expression of these isoforms is therefore one advantage of the longer 26 bp tags.

However, also methodological inconsistencies may result in the observation of several different tags from a single gene [25]. To prevent methodological artefacts and assure the validity of the detected transcript variants, confirmative procedures such as double *Nla*III digestion were standard in all libraries. Additionally, *in silico *routines for the exclusion of artefacts were applied (e.g. elimination of twin ditags and singletons [7]).

In the following sections, we discuss the expression of only a subset of drought-regulated genes and gene family members. We focussed on genes known to be involved in stress-perception, signalling and transcription initiation, because at least some of them are well characterized, and often clear-cut evidence is available for their role in stress-responses [3]. As has been shown for tolerance to salt-stress in rice, these genes or their products regulate early events in drought responses, differentiating between stress-tolerant and -susceptible genotypes [26]. However, genes involved in stress perception and signalling are not necessarily most up- or down-regulated.

### Stress perception and signalling in drought-stressed chickpea roots

Dehydration-related stresses such as drought and salinity have ionic as well as osmotic attributes that elicit signal transduction cascades resulting in activation of effector genes to adapt the metabolism of the plant to the stress. In the model arising from research in *Arabidopsis *or rice, the first step of signalling is the perception of the stress through G-protein coupled receptors (GPCR), inositol polyphosphates, or receptor-like kinases (RLKs;[2]). In drought-stressed chickpea roots, 36 RLK transcript variants were detected. One of these increased in abundance more than 20-fold, whereas fourteen were 2 to 8-fold up-regulated under stress, indicating a potential role of these transcripts in stress perception. Calcium transients are major signalling events in plants [27]. Thus, entry points and interconnecting links in major stress-related signalling cascades involve Ca^2+ ^sensors and proteins regulated by Ca^2+^-concentration gradients between apoplast and cytoplasm [28]. In drought-stressed chickpea roots, SuperSAGE revealed moderate to significant changes in expression levels of Ca^2+^-responsive genes. Transcript isoforms encoding Ca^2+ ^sensors (e.g. calcineurin-B-like proteins, CBLs), Ca^2+^-channel proteins, and inositol-3-phosphate (IP_3_)-gated Ca^2+^-release (e.g. phospholipase C), were generally up-regulated (for an extensive characterization see [29,30]). In contrast, transcript isoforms transcribed from genes involved in downstream events like fine-tuning of, and interconnecting between, signalling cascades were up- as well as down-regulated in reaction to the stress. These included transcripts encoding a wide range of kinases such as Ca^2+^-dependent protein kinases (CDPKs; [31]), calcineurin-B-interacting protein kinases (CIPKs, [32])., and protein phosphatases class 2C.

Since the interplay between kinases and phosphatases balances activation and inactivation of proteins and with it the cross-talk between signaling cascades and metabolic pathways [33], measurement of transcriptional activity of kinase-encoding genes is important for understanding drought-response homoeostasis. Mitogen-activated protein kinases (MAPKs), however, do not seem to interact much with early drought-stress signalling in chickpea roots, since of 6 MAPK transcripts detected, 2 were down-regulated more than 8-fold, and 4 similarly expressed as in control roots. In addition, the only MPKK detected was constitutively expressed, whereas from three 26 bp tags annotated to MPKKKs, two were down-regulated at least 4-fold (STCa-8893, STCa-10844), and one transcript was up-regulated at least 6-fold (STCa-2124).

### Regulation of 14-3-3 transcripts

Signal transduction and regulation of metabolism achieved via phosphorylation-mediated transition of protein states require that phosphorylated proteins physically interact with specialized adapter proteins to fulfil their regulatory role. An example for such adapters are the phosphoserine/threonine-binding 14-3-3 proteins [34]. For example, 14-3-3 proteins are phosphorylation targets for SnRK 2.8, a member of the sucrose non-fermenting-related kinase family, that is down-regulated in plants deprived of nutrients and with reduced growth [35]. Plants have large 14-3-3 gene families, and various 14-3-3 isoforms have varying affinities to target proteins. In *Arabidopsis*, at least 15 expressed members of the 14-3-3 gene family exist and exhibit high cell- and tissue-specificity as well as diverse expression levels [36]. In rice, at least four 14-3-3 transcript isoforms are induced by drought and salt-stress [37].

In drought-stressed chickpea roots, we detected 18 isoforms of 14-3-3 protein transcripts. However, contrary to results from drought-stressed rice, only three of these were up-regulated more than 2-fold, whereas ten were down-regulated more than 4-fold (Figure 5). Similar differential expression of 14-3-3 transcript isoforms was detected in young tomato roots under normal growth conditions as well as in response to salt stress and potassium and iron deficiencies, suggesting that especially one isoform (TFT7) may mediate cross-talk between the salt stress and potassium and iron-deficiency signalling pathways, respectively [38].

### Transcription factors and involvement of ABA in early drought stress responses in chickpea roots

**Figure 10:**
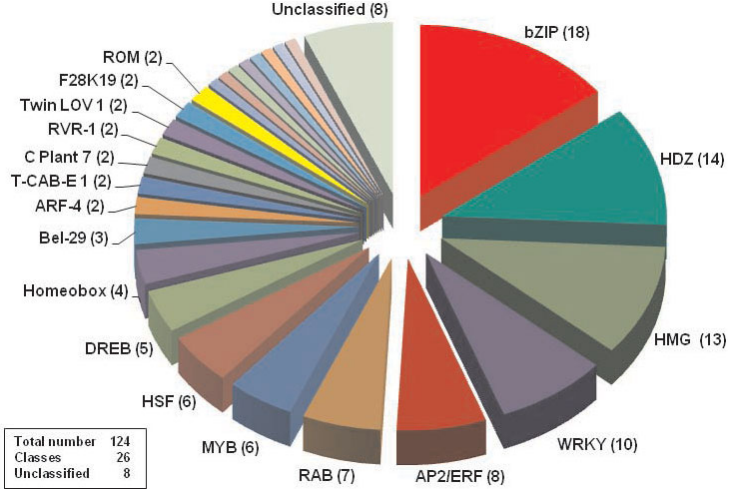
**Transcription factor classes in SuperSAGE libraries from chickpea roots**. Transcription factor classes in SuperSAGE libraries from drought-stressed chickpea roots. Numbers in parentheses represent the number of 26 bp tags annotated to each class.

Another large and complex class of genes encode transcription factors (TFs). We could identify 124 UniTags from TF transcripts classified into 26 TF families (Figure 10), whereas 8 remained un-classified. The majority of TF-UniTags annotated to the bZIP TF family (18), followed by UniTags matching to the HDZ (14), HMG (13), and WRKY (10) TF families. As described for the bZIP type TF family in Figure 5, expression profiles of transcription factors-encoding transcripts may display diverse regulation tendencies. This is also true for UniTags derived from transcripts encoding MYB family members, though these were reportedly involved in signal transduction under water-deficit [39]. This observation may be related to the constitutive and even slight down-regulation of transcripts for proteins involved in ABA synthesis such as 9-*cis-*epoxy-carotenoid dioxygenase [40], though Boominathan and co-authors [5] observed a strong up-regulation of a particular mRNA for this enzyme under drought stress, that probably escaped our detection. The difference in expression of genes involved in ABA-dependent signalling such as the isoforms of MYB TFs [41] may suggest that, as in maize, ABA as signal may be restricted to very specific regions of the root [42]. From six MYB transcription factors detected, two were up-regulated, and 3 were down-regulated. Of 18 members of the bZIP TF family (see above) to which AREB factors belong [43], four were up-regulated more than 3-, whereas three were down-regulated more than 4-fold.

In contrast, ABA-independent signalling seems to prevail: two out of five Drought-Responsive-Element-Binding (DREB2) TF isoforms thought to be regulated independently from ABA [44] were at least 3-fold up-regulated (STCa- 4170, STCa- 4212). Additionally, two further DREB transcript variants revealed constitutive levels, whereas one was 3-fold down-regulated (STCa-13360). Besides DREB2 TFs, WRKY TFs also seem to be involved in drought-stress responses in chickpea, because of the 10 detected family members, two were at least 2-fold (STCa-4132, STCa-10200), and two 4-fold up-regulated (STCa-11618, STCa-11619). Also, UniTag STCa-15340, homologous to an alfin-1-like TF transcript from alfalfa, belonged to the 40 most up-regulated transcripts under drought stress in chickpea (Table 4). In alfalfa, transgenic over-expression of a TF belonging to this class enhances expression of the endogenous *MsPRP2 *gene and improves salinity tolerance [45]. Thus, up-regulation of STCa-15340 indicates the involvement of alfin-1-like TFs in salt- as well as in drought-stress responses. Though already highly informative, our analysis of TF gene expression in drought-stressed chickpea roots certainly tapped the tip of an iceberg only, since we could assign only 124 UniTags to this class of genes. In fact, there are more than 1500 TF genes in the *Arabidopsis *genome [46]. Considering that we could assign only 22% of the 26 bp tags to well characterized entries in the public data bases, the number of TFs in our data set should be approx. 5 to 6 times as high as the one we could assign, i.e. approx. 650. This estimate is in good agreement with results from sugarcane, where 237,954 ESTs contained 600 TF sequences [47].

### ROS scavenging and ROS-triggered signalling-related genes

Plants generate singlet oxygen-, superoxide-, peroxide-, and hydroxyl-radicals (ROS) that trigger a wide range of partly genetically fixed responses. ROS are released by either NADPH oxidases or peroxidases, that may exist alone or in combination in different plant species. Plant cells perceive changes in the concentrations of ROS as second messengers, and transform them into signals that change the transcription of genes [2]. On the other hand, disturbances in metabolism and photosynthesis by environmental stresses lead to ROS accumulation, which, if not controlled, can rapidly reach toxic levels in the plant cell [48]. Major ROS scavenging enzymes include superoxide dismutase (SOD), ascorbate peroxidase (APX), mono-dehydroascorbate reductase (MDAR), dehydroascorbate reductase [49], glutathione-S-transferase (GST) [50], glutathione peroxidase (GPX), glutathione reductase (GR), and catalase (CAT) [51]. The balance between the activities of these enzymes could be crucial for determining the steady-state level of ROS.

Our data reflect the complexity of ROS signalling and scavenging. For example, 10 out of 29 peroxidase isoforms were significantly up-regulated (data not shown), but only one NADPH oxidase was detected and moderately up-regulated. However, one transcript each for a hypersensitive-induced response protein and radical-induced cell death 1-1 protein were detected, and both were significantly up-regulated. Of the ROS scavengers, six out of seven SOD transcripts, and one out of two transcripts coding for each catalase and dehydroascorbate reductase were more than 2-fold up-regulated, whereas two of the three ascorbate peroxidase (APX) transcripts were moderately or even significantly down-regulated. Since APX catalyses the H_2_O_2_-dependent oxidation of L-ascorbate (vitamin C;[52]), this potent ROS scavenging mechanism seems to be under-used in early responses of chickpea roots to drought. Considering that STCa-7166 representing an NADP-dependent isocitrate dehydrogenase transcript belonged to the most up-regulated transcripts in our study (R_(ln) _3.58, Table 4), scavenging of ROS by gluthatione and recycling of oxydized gluthatione by this enzyme seems to prevail instead. This assumption is supported by the strong up-regulation (R_(ln) _3.08, Table 4) of cysteine synthase (CS)-encoding UniTag STCa-2982. In rice, as a consequence of CS up-regulation, both the total glutathione pool and reduced glutathione concentration were significantly increased in response to aluminium stress [53].

Still another stress-responsive gene family encodes glutathione-S-transferases (GSTs), key defence enzymes against xenobiotic toxicity, and has at least 56 members in rice [54]. From 14 chickpea 26 bp tags annotated to GSTs, four isoforms (STCa-977, 2175, 20830 and 12384) were at least 2-fold down-regulated, and three isoforms revealed up-regulation (STCa-3042, 12502 and 22470). Apart from ROS-scavenging, GSTs may also function in stress tolerance through signalling [55]. Considering the importance of these proteins for managing ROS-related stress, the GST transcript variants strongly up-regulated in chickpea roots under drought stress are potential targets for molecular breeding for drought tolerance.

### Regulation of aquaporin gene activity under drought stress

Despite considerable progress in understanding fundamental stress responses in model plants, we know little about the molecular basis of differences between stress-tolerant and susceptible genotypes of crops. Only recently, investigations into drought responses of upland and lowland rice, and genotypic variation for water status under different water regimes in a population of recombinant inbred lines (RILs) of sunflower [56] demonstrated a possible involvement of certain aquaporin genes in differing dehydration-stress response phenotypes of these crops. For example, the *Arabidopsis *genome harbours at least 35 genes coding for different aquaporins, that are differentially expressed under different stress conditions and, whereas one family member is up-, the other may well be down-regulated. In chickpea roots, we detected at least 42 different 26 bp tags from aquaporin transcripts, representing the three classes (nodulin-, plasma membrane-, and tonoplast-intrinsic forms). Interestingly, the over-expression of a certain aquaporin isoform in transgenic *Arabidopsis *resulted in altered expression patterns of other aquaporin isoforms with consequences for seed germination, seedling growth, and stress responses of the plants under various stress conditions [57]. These results suggest a concerted transcriptional regulation of at least a subset of aquaporin genes. Like in *Arabidopsis, *drought also elicited differential responses in the different members of the aquaporin gene family also in chickpea. It will be interesting to learn, whether the differentially expressed putative NATs corresponding to several of the sense aquaporin isoforms detected in chickpea are involved in the regulation of other gene family members. Also, whether differences exist in the expression of aquaporin isoforms between drought-tolerant and -susceptible genotypes of chickpea needs to be determined.

### Changes in transcription of genes regulating compatible osmolyte accumulation

Beyond the broad repertoire of signalling cascades and signalling interactions that plants have on their defence lines against drought stress, the accumulation of sugars, sugar alcohols, amino acids, and polyamines, acting as compatible osmolytes against the osmotic disequilibrium is one of the most widespread strategies of plants to enhance their tolerance against drought stress [58]. Several genes involved in biosynthesis, transport as well as intermediate and catabolic pathways related to this strategy have been genetically and functionally characterized during the past years [59-67]. After screening the *C. arietinum *UniTags database for the behaviour of genes related to the above processes, transcription profiles and number of isoforms from at least 12 relevant genes were investigated in detail (Figure 6). Related to sugar accumulation, one UniTag annotated to trehalose-6-phosphate synthase (STCa-18759, 2-fold down-regulated), and three 26 bp tags annotated to trehalose-6-phosphate phosphatase (STCa-9149 3-fold upregulated; STCa-11438 3-fold down-regulated; STCa-21065 constitutive) were observed. Trehalose plays an important role as compatible osmolyte and signalling molecule under drought stress [60,62]. However, since we do not observe strong up-regulation of genes encoding threhalose-6-phosphate synthase, we are hesitant to conclude that threhalose accumulated to high concentrations under our assay conditions. Additionally, one significantly up-regulated UniTag STCa-11968 representing a galactinol synthase gene as well as at least three tags representing transcripts related to sucrose metabolism and transport with more than 2-fold expression changes (STCa-19100, STCa-8449, Ca-SS-16426) revealed, that the dynamics of sugar metabolism, transport and accumulation could be altered as a response to drought stress in chickpea. The positive role of galactinol synthase in stress tolerance has already been reported elsewhere [64]. Several 26 bp tags representing amino acid transport- and accumulation-related genes were detected. For proline, a compatible osmolyte [67], one 6-fold up-regulated UniTag with homology to a proline/betain transporter (STCa-24308) as well as moderate down- and up-regulation of two 26 bp tags representing a negative regulator for proline accumulation (proline dehydrogenase; STCa-8454, STCa-8455) were revealed. This suggests that prolin accumulation may occur to some extent under drought stress in chickpea. The transcript levels for betaine aldehyde dehydrogenase UniTag STCa-14752, the key enzyme for glycine betaine synthesis [68], did not markedly change upon drought stress, indicating that this osmolyte, otherwise reported to accumulate under water stress [69], is not important for an early stress response. Since the accumulation of polyamines as compatible osmolytes is discussed as protection against stress, we paid special attention to transcripts encoding arginine decarboxylase and spermidine synthase, that share important roles in putrescine and spermidine accumulation [70]. The detected transcripts were 2-fold (STCa-8875; arginine decarboxylase) and 3-fold (STCa-611; spermidine synthase) up-regulated. Our results indicate a differential influence of drought stress on mechanisms for compatible osmolyte accumulation as an early stress response in chickpea.

### Comparison of SuperSAGE versus macro- and micro-arrays

Though drought-stress responses from roots of adult chickpea plants were compared to the roots of much younger seedlings from *Medicago *([10], Table 7), the similar expression of several genes (section D, Figure 9) suggests similar reactions of the roots of both legumes to drought stress, independently of the developmental stage of the roots. Genes similarly up-regulated in both species *inter alias *include a certain extensin isoform, actually the most up-regulated transcript in stressed chickpea roots (O65760_CICAR). Extensins are hydroxyproline-rich proteins strengthening cell walls, and are often activated by mechanical stress [71]. Like other proline-rich cell-wall proteins, extensins require hydroxylation of prolines to 4-hydroxyprolines to form the cell wall matrix [72]. Consistent with extensive hydroxylation of extensin, UniTag STCa-542 representing a prolyl 4-hydroxylase alpha subunit-like protein belonged to the most up-regulated transcripts in drought-stressed chickpea roots (R_(ln) _2,722).

At the same time, STCa-1804, encoding an expansin-like protein, was most down-regulated (R_(ln) _-3,095) in stressed chickpea roots. Expansins weaken cell walls [73], and thus down-regulation of the expansin gene also would contribute to an increased strength of the cell walls of drought-stressed roots. In addition, UniTag STCa-24349, representing a gibberellin 2-beta- hydroxylase transcript, belonged to the most up-regulated transcripts in stressed chickpea roots (R_(ln) _2,28, Table 4). The encoded enzyme inactivates gibberellin through β-hydroxylation [74] and thus, its up-regulation should result in significantly deceased levels of bioactive hormone and a reduction of cell divisions and extensions. We conclude that in chickpea and *Medicago*, drought impairs division and extension of cells and results in growth-retarded roots with strengthened cell walls.

Other transcripts similarly up-regulated in both chickpea and *Medicago inter alias *code for dehydrin, DREB, 2C protein phosphatase, UDP-glucose phosphorylase and blue copper protein which are discussed elsewhere in this paper. Taken together, a comparison of drought stress responses in chickpea and *Medicago *reveals many inter-species similarities and suggests to exploit the huge resources available for *Medicago *to test the functions of differentially expressed transcripts in chickpea.

## Conclusion

Applying SuperSAGE to the analysis of abiotic stress responses in chickpea for the first time, our study presents the most comprehensive transcriptome profile of this crop available to date. It increases the number of chickpea ESTs from approximately 1,900 to more than 80,000, and the number of unique transcripts to more than 17,000. The study identified major drought-stress signalling cascades resulting in differential expression of effector genes, and hints to the importance of ROS and N starvation as side stresses resulting from drought. Our study revealed, that (1) genes involved in photosynthesis and energy metabolism were down-regulated, (2) many genes involved in early responses to biotic and abiotic stresses were up-regulated, while (3) many other stress-responsive genes were down-regulated, and (4) regulatory genes encoding e.g. transcription factors or signal transduction proteins were both up- and down-regulated. We conclude that follow-up transcription profiling studies of responses to drought in chickpea must take into account the potentially deleterious effect of the stress on SNF and thus, on N supply to the plant in order to prevent mixing up responses to different stresses.

One important fact arising from our study is the unexpectedly high number of differentially expressed isoforms of members of large gene families, that was also observed in SAGE libraries from *Lotus japonicus, *where different levels of transcription induction among leghemoglobin gene paralogs were found [12]. These findings highlight the efficiency of tag-based techniques to discriminate different gene family members. At the same time, they underpin the necessity to – experimentally and linguistically – distinguish between certain transcript isoforms (and the underlying genes) rather than summarizing them under a common term.

With this work, we aimed at identifying candidate genes as targets for molecular breeding for drought tolerance in chickpea. Numerous studies confirmed the polygenic nature of drought tolerance, for which single QTLs have only little individual effect. Considering the large number of genes located at QTLs for drought tolerance and related traits in cereals, comparing our transcription profiles to genes mapped to drought QTLs in these crops may help to decide whether SuperSAGE has identified such potential breeding targets.

## Methods

### Plant materials and stress treatment

Surface-sterilized seeds of drought-tolerant chickpea variety ILC588 (Rehman et al. ICARDA, ) were germinated in germination boxes on filter paper at ICARDA (Syria). The resulting seedlings were grown in a growth chamber at a constant temperature of 22°C, a photoperiod of 12 h light/12 hours dark and normal watering. After eight days, the seedlings were transferred onto composite soil for a hardening period of 20 days at 20 – 25°C during day/15 – 20°C during night with a photoperiod of 16 hours light and 8 hours dark. Then control plants were removed, and their roots immediately frozen in liquid nitrogen. For desiccation, plants were removed, carefully preventing mechanical damage, and subjected to dehydration for 6 h at room temperature. Light regime, temperature, and humidity were kept constant and strictly monitored during the treatment of the plants. After the desiccation period, the plants showed wilting symptoms (turgor loss), and the roots were separated from the shoots and shock-frozen in liquid nitrogen.

### RNA isolation and construction of SuperSAGE libraries

Total RNA was isolated from control and stressed roots using a modified CTAB procedure [75] followed by precipitation of the RNA in 3 M LiCl at 4°C overnight. From approximately 1 mg of total RNA, poly(A)^+^-RNA was purified using the Oligotex mRNA Mini Kit (QIAGEN, Hilden, Germany) according to the manufacturer's batch protocol. Subsequent steps for construction of SuperSAGE libraries were performed as detailed by [76]. However, instead of concatenation of di-tags and subsequent cloning and sequencing, amplified ditags were directly sequenced by 454 Life Sciences, Branford, CT, USA.

### Tags quantification and data analysis

For each library, 26 bp long 26 bp tags were extracted from the sequences using the GXP- Tag sorter software provided by GenXPro GmbH, Frankfurt am Main, Germany. Library comparison and primary statistical treatment was carried out using the DiscoverySpace 4.01 software (Canada's Michael Simith Genome Sciences Centre, available at ). Scatter plots of the distribution of the expression ratios (R_(ln)_) and significance of the results were calculated according to Audic and Claverie [11].

### Sequence homology alignment

Tags sequences were BLASTed [77] against different public databases discriminating the hits in a hierarchical, taxonomical manner using the BLASTN algorithm . First, all 17,493 unique tag sequences were BLASTed against the non-redundant DNA databases, limiting the output hits with the highest priority level to *Cicer arietinum *and members of the Fabaceae, by using the routine BLASTc13 (NCBI, ). Subsequently, individual local BLAST searches were carried out in Fabaceae sequences, followed by *Arabidopsis*, rice and maize homology searches in the TIGR gene indices . After each BLAST round, anonymous DNA sequences (e.g. chromosomes, shotgun clones, and ESTs not linked to any characterized protein) were filtered out. Additionally tags assigned to TIGR TCs indicating weak similarity to characterized genes were not selected. For targets from legumes different from chickpea, a maximum of three mismatches was allowed. The expected number of random matches (E value) was kept under 0.009 for individual TIGR databases, and 0.0009 for larger databases (e.g. NCBI nr restricted to fabaceae hits). Low complexity regions were rejected, whereas gap costs were set to 5-2 (NCBI BLAST standard setting).

### Annotation test of *in silico *generated chickpea 26 bp tags using *M. truncatula *ESTs

In order to test the validity of the annotation of chickpea tag sequences through homologies with other legumes, 7,500 chickpea EST sequences deposited in the NCBI data bank were used to generate virtual 26 bp tags. Initially, all ESTs were screened for CATG sites using the BioEdit software, version 7.0.5.3 . Subsequently, all ESTs harbouring more than 30 bp between the most 3' CATG site and the end of the sequence were selected. After virtual 26 bp tag extraction, duplicate fragments were excluded, and the remaining tags BLASTed against public EST/mRNA databases following three main routes: I) BLASTing against the non-redundant (nr) NCBI nucleotide database (*Fabaceae *mRNA accessions), II) against the plant EST database at NCBI (*M. truncatula *accessions), and III) against *M. truncatula *ESTs deposited in the TIGR gene indices. Complete EST/mRNA high homology target sequences derived from BLASTs (II) and (III) were retrieved and reBLASTed against the nr NCBI database (*Fabaceae*). The results obtained by these two BLAST strategies were compared with strategy (I) after exclusion of anonymous entries (e.g. AFLP fragments, shotgun sequencing clones, whole genome entries, whole chromosomes, BAC clones, etc.). BLAST parameters were set as described in the previous section.

### Cluster analysis and functional category distribution analysis

Cluster analysis of the expression ratios (R_(ln)_) used the software package Cluster 3.0 . A distance matrix for the R_(ln) _was calculated with Pearson's correlation distance method [78]. Transcripts were clustered using the average linkage clustering routine under hierarchical clustering. P values for the most represented GO: biological processes observed after 6 hours of desiccation were calculated and correlated with the UniTag expression ratios (R_(ln)_) using the "Receiver Operator Characteristic" (ROC) routine of the ermineJ 2.0 software package (University of British Columbia, 2006, . P-values for the representation of GO: categories are calculated according to [79] as indicated by the software developers.

### Rapid amplification of cDNA ends (3'-RACE) using tag sequences as PCR primers

To test the versatility of the 26 bp tag-derived oligonucleotides for direct use as 3'-RACE PCR primers, cDNA amplifications were carried out with an initial denaturation step of 94°C for 2 min, followed by 30 cycles each of 94°C for 40 sec, 55°C for 1 min, and 72°C for 1 min, with a final extension step at 72°C for 4 min. Reactions contained 15–20 ng cDNA template, 10 pmol 26 bp tag-based primer, 10 pmol oligodT (t)14-NV primer, 200 μM dNTPs, 0.4 U *Taq *DNA polymerase (Genecraft, Germany) in buffer containing 1.5 mM MgCl_2 _supplied by the provider. After amplification, products were separated in 1.5% preparative agarose gels. Bands corresponding to unequivocal amplicons were excised, and DNA extracted with Qiaquick cleanup columns (QIAGEN, Hilden, Germany). Cloning of PCR products as well as colony PCR screening followed stanrdard blue-white screening procedures [80]. Positive clones were sequenced via ABIprism multi-colour fluorescence-based DNA analysis system (APPLIED BIOSYSTEMS, Foster City CA, USA).

### Confirmation of SuperSAGE expression profiles via qRT-PCR

Parallel RNA extractions of the same tissue, from which the SuperSAGE libraries were derived, were carried out as described in a previous section. Approximately 500 ng of total RNA were further processed to poly(A)^+^-RNA via Oligotex matrix (QIAGEN, Hilden, Germany). cDNA was synthesized using the Superscript III double-stranded cDNA synthesis kit (INVITROGEN, Karlsruhe, Germany). Resulting cDNA was quantified with two parallel methods: i) Nanodrop spectrometer measurement (NANODROP, Willmington DE, USA, and ii) Caliper chip quantification (CALIPER, Hopkinton MA, USA).

SYBR green oligonucleotide deduction was carried out with the software package Primer Express, version 2.0, provided by Applied Biosystems (Foster City, CA, USA) with 3'- or 5'-RACE products from selected 26 bp tags as starting points. The two *Taq*Man assays used in this study were provided by GenXPro GmbH, Frankfurt, Germany, and used according to the protocol included in the kit.

The real-time PCR reactions for SYBRgreen and *Taq*Man assays used the Power-SYBRgreen PCR master mix and the *Taq*Man-Universal PCR Master mixes, respectively (Applied Biosystems). RT-PCR amplifications were carried out in a StepOne RT-PCR System machine with the following temperature profile for SYBRgreen assays: initial denaturation at 95°C for 10 min, followed by 40 cycles of 95°C for 10 sec. and 60°C for 20 sec. (annealing and elongation). *Taq*Man assay temperature amplification profiles consisted of an initial denaturation at 95°C for 10 min, followed by 40 cycles of 95°C for 10 sec. and 65°C for 30 sec. Amplicon quality was checked by an additional melting curve gradient with fluorescence measures after each temperature step. The amplification of the target genes at each cycle was monitored by SYBRgreen- or *Taq*Man probe-released fluorescence. The Ct, defined as the PCR cycle at which a statistically significant increase of reporter fluorescence is first detected, was used as a measure for the starting copy numbers of the target gene. Relative quantitation of the targets amplified via SYBRgreen assays was performed by the comparative ΔΔCt method. Genes amplified by *Taq*Man assays were quantified via the Relative Standard Curve Method (Applied Biosystems). The efficiency of each primer pair was checked with cDNAs from control and 6h-desiccation as standard templates. The RT-PCR data were normalized with the relative efficiency of each primer pair.

### Confirmation of expression profiles via microarrays

SuperSAGE expression profiles were confirmed by direct spotting of a selection of 26 bp tags onto a 16 K Agilent microarray (AGILENT TECHNOLOGIES, Santa Clara CA, USA). Three thousand UniTags with different expression levels under drought, salt, and cold stresses (salt and cold stress expression profiles are not approached in the present paper) were selected. From the 3,000 Tags, a subset of 2,796 oligonucleotides was spotted in duplicate onto different sections of the microarray. Additionally, for each of the 3,000 selected Tags, oligonucleotides with mismatches were spotted onto the microarray in three sets as follows: i) mismatch at position 7; ii) mismatches at positions 7 and 13 and iii) mismatches at positions 7, 13, and 20, respectively. Background correction was achieved by the Feature Extraction softwareTM (Agilent Technologies), subtracting the mismatch intensities for each spotted Tag. Microarray design, spotting and hybridizations were carried out by ARRAY-ON GmbH, Gatersleben, Germany, according to the AgilentTM protocols (AGILENT TECHNOLOGIES, Santa Clara CA, USA).

## Authors' contributions

CM and BR generated the SuperSAGE libraries with the guidance of HM and RT and performed in silico data analyses. RH generated the 3-' and 5'-RACE sequences and designed the primers for qRT-PCR probes. MB and SMU selected the plant material, performed the stress treatments and provided the root material. BB and LB programmed and applied bioinformatic tools for analysis of large sets of SuperSAGE tags. GK and PW developed the experimental strategy, and were responsible for the preparation of the manuscript.

## Supplementary Material

Additional file 1 **Main data matrix** Click here for file

Additional file 2 Chickpea in silico tags annotation Click here for file
